# Alteration of protein function by a silent polymorphism linked to tRNA abundance

**DOI:** 10.1371/journal.pbio.2000779

**Published:** 2017-05-16

**Authors:** Sebastian Kirchner, Zhiwei Cai, Robert Rauscher, Nicolai Kastelic, Melanie Anding, Andreas Czech, Bertrand Kleizen, Lynda S. Ostedgaard, Ineke Braakman, David N. Sheppard, Zoya Ignatova

**Affiliations:** 1 Biochemistry, Institute of Biochemistry and Biology, University of Potsdam, Potsdam, Germany; 2 School of Physiology, Pharmacology and Neuroscience, University of Bristol, Bristol, United Kingdom; 3 Institute for Biochemistry and Molecular Biology, Department of Chemistry, University of Hamburg, Hamburg, Germany; 4 Cellular Protein Chemistry, Department of Chemistry, Bijvoet Center for Biomolecular Research, Utrecht University, Utrecht, The Netherlands; 5 Department of Internal Medicine, University of Iowa, Iowa City, Iowa, United States of America; University of Bath, United Kingdom of Great Britain and Northern Ireland

## Abstract

Synonymous single nucleotide polymorphisms (sSNPs) are considered neutral for protein function, as by definition they exchange only codons, not amino acids. We identified an sSNP that modifies the local translation speed of the cystic fibrosis transmembrane conductance regulator (CFTR), leading to detrimental changes to protein stability and function. This sSNP introduces a codon pairing to a low-abundance tRNA that is particularly rare in human bronchial epithelia, but not in other human tissues, suggesting tissue-specific effects of this sSNP. Up-regulation of the tRNA cognate to the mutated codon counteracts the effects of the sSNP and rescues protein conformation and function. Our results highlight the wide-ranging impact of sSNPs, which invert the programmed local speed of mRNA translation and provide direct evidence for the central role of cellular tRNA levels in mediating the actions of sSNPs in a tissue-specific manner.

## Author summary

Synonymous single nucleotide polymorphisms (sSNPs) occur at high frequency in the human genome and are associated with ~50 diseases in humans; the responsible molecular mechanisms remain enigmatic. Here, we investigate the impact of the common sSNP, T2562G, on cystic fibrosis transmembrane conductance regulator (CFTR). Although this sSNP, by itself, does not cause cystic fibrosis (CF), it is prevalent in patients with CFTR-related disorders. T2562G sSNP modifies the local translation speed at the Thr854 codon, leading to changes in CFTR stability and channel function. This sSNP introduces a codon pairing to a low-abundance tRNA, which is particularly rare in human bronchial epithelia, but not in other human tissues, suggesting a tissue-specific effect of this sSNP. Enhancement of the cellular concentration of the tRNA cognate to the mutant ACG codon rescues the stability and conduction defects of T2562G-CFTR. These findings reveal an unanticipated mechanism—inverting the programmed local speed of mRNA translation in a tRNA-dependent manner—for sSNP-associated diseases.

## Introduction

Synonymous single nucleotide polymorphisms (sSNPs) in protein-coding regions occur at much higher frequency in the human genome [1] than initially assumed. Owing to the degeneracy of the genetic code (that is more than 1 codon specifying 1 amino acid), sSNPs are considered silent or invariant for protein folding and function as they synonymously exchange only codons, but not the encoded amino acids. As a corollary of this view, sSNPs have been rationalized as neutral for selection and fitness of an organism [2]. However, synonymous codons of an amino acid are not equally used and the bias in codon usage suggests that synonymous codons have been under evolutionary pressure [3]. Natural selection of rarely used codons conditions circadian rhythm–dependent gene expression [4, 5] and synchronizes mRNA translation with downsteam processes, including protein folding and translocation [6–8]. Thus, sSNPs that alter codon usage might affect cotranslational protein folding and protein conformation. Furthermore, sSNPs might alter local mRNA secondary structure [9], binding sites for RNA-binding proteins or regulatory miRNAs, thus also impacting physiological function [10]. So far, the little experimental evidence in eukaryotic systems linking sSNPs with conformational changes in proteins [11, 12] lacks mechanistic explanation.

Genome-wide association studies link sSNPs with ~50 diseases in humans, highlighting the wide-ranging impact of SNPs that exchange synonymous codons; however, their association with alterations in protein conformation and function remains elusive [10]. Conceptually, codon usage is considered as a proxy for the speed each codon is translated: rare codons are more slowly translated than abundant codons [13]. However, global analysis of ribosome progression along mRNAs in mammalian cells argues that rare codons do not always have an effect on translation speed [14, 15]. A major determinant of elongation speed for a codon is the concentration of its cognate tRNA [16] and the ratio of cognate to near-cognate tRNA [17]. In prokaryotes and unicellular eukaryotes, tRNA concentration correlates well with codon usage [18]. By contrast, in mammalian systems, tRNA concentration varies between proliferating and differentiating cells [19], among organs [20], and strikingly, some tRNAs are uniquely expressed in defined subregions of one organ [21]. These data argue that the rate of translation of a codon cannot be determined simply from genome usage. They also suggest that in multicellular eukaryotes, codon translation speed might vary among different cells and tissues despite uniform genomic codon usage. Hence, the effects of sSNPs might be restricted to specific tissues and are not predictable from codon usage. Consequently, some sSNPs might have so far escaped detection, and their precise effects on protein function remain unresolved, with potential implications for human health.

Here, we investigated the impact of sSNPs on the cystic fibrosis transmembrane conductance regulator (CFTR). CFTR is an ATP-binding cassette (ABC) transporter that functions as a ligand-gated anion channel [22]. Dysfunction of CFTR causes the common, life-shortening disease cystic fibrosis (CF) [23]. Although the ΔF508 mutation is by far the most prevalent CF mutation, to date, more than 2,000 different mutations have been found in the *CFTR* gene, which vary in disease severity and penetrance [23]. Despite major advances in classifying mutations within the CFTR gene [24], the mechanisms of dysfunction of the large majority, including almost 270 SNPs (comprising both synonymous and nonsynonymous nucleotide substitutions), remain enigmatic. We integrated global analyses of tRNA concentration and translation with (i) thermal stability and proteolytic susceptibility assays as a reporter of CFTR conformation and (ii) single-molecule activity measurements as a readout of CFTR function to comprehensively determine the effects of sSNPs on CFTR expression and function. We identified an sSNP that inverts the programmed local speed of mRNA translation in a tissue-specific, tRNA-dependent manner to alter the global conformational dynamics and physiological function of CFTR.

## Results

### The T2562G sSNP reduces the amount of CFTR protein

**Fig 1:**
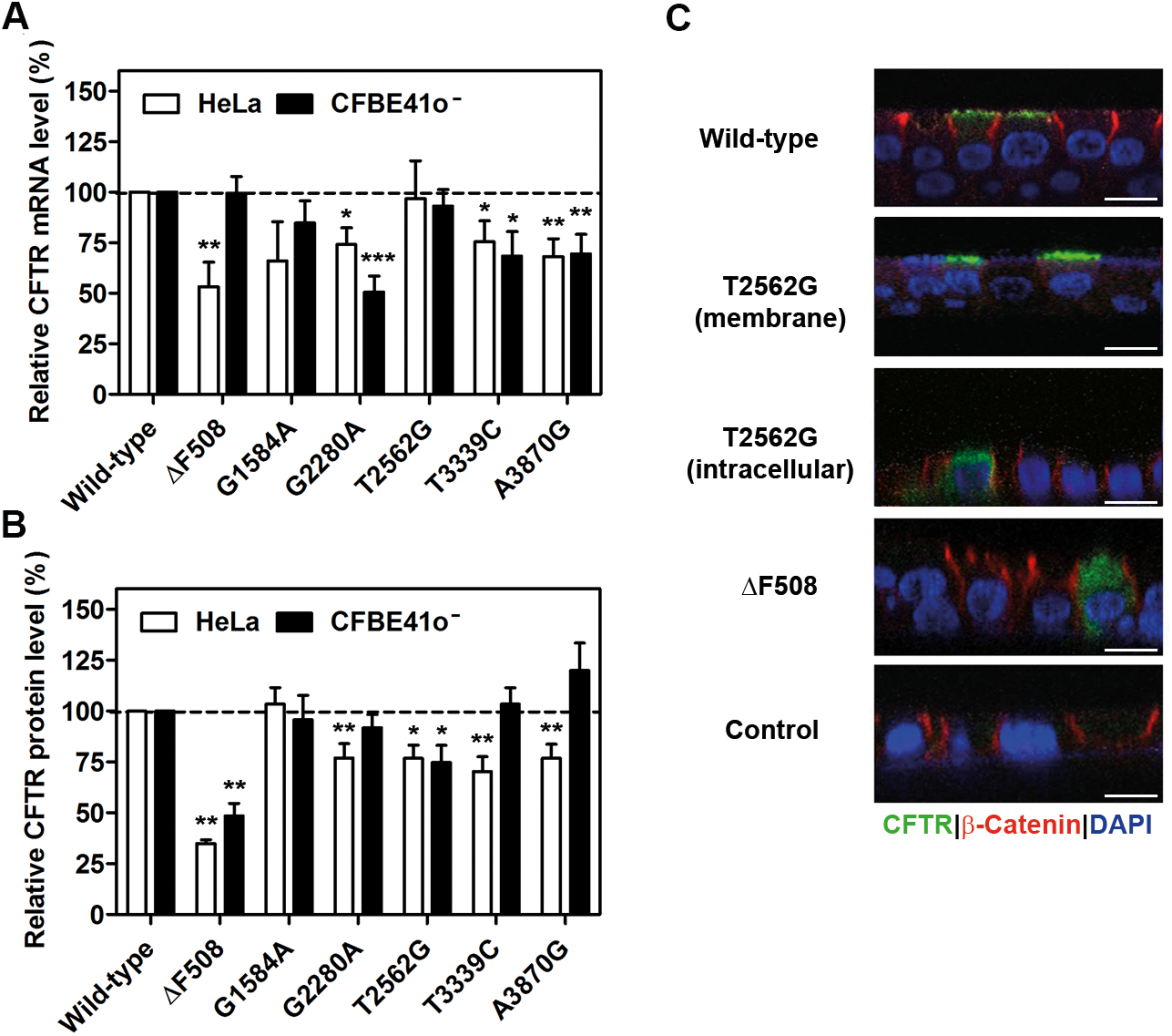
Synonymous mutations alter cystic fibrosis transmembrane conductance regulator (CFTR) expression. (**A**) Quantitative real-time PCR (qRT-PCR) quantification of steady-state CFTR mRNA expression in HeLa and CFBE41o^-^ cells analyzed 24 h after transfection and normalized to neomycin phosphotransferase (NPT). The mRNA level of wild-type CFTR was set to 100%. (**B**) Total steady-state CFTR expression (i.e., the sum of bands B and C in S1B Fig) normalized to that of NPT and β-actin (ACTB) and related to wild-type CFTR, which was set to 100%. In A and B, data are means ± SEM (A: HeLa, *n* = 3–7; CFBE41o^-^, *n* = 4–9; B: HeLa, *n* = 5–14; CFBE41o^-^, *n* = 4–7); * *P* < 0.05, ** *P <* 0.01, *** *P* < 0.001 versus wild-type CFTR. (**C**) Representative immunostaining images (*n* = 3) of well-differentiated human cystic fibrosis (CF) airway epithelia. The frequency of intracellular versus membrane-localized T2562G-CFTR staining was as follows: 44% of T2562G-CFTR–expressing cells exhibited intracellular staining (12 out of 27) and in 55% (15 out of 27), staining was membrane localized. The perinuclear staining demonstrates the intracellular localization of T2562G-CFTR. The control was non–CFTR-expressing epithelia. β-catenin immunostaining denotes adherens junctions and DAPI—nuclei; scale bars, 10 μm. The underlying data of panels A and B can be found in S1 Data.

From all ~270 polymorphic mutations in the *CFTR* gene (http://www.genet.sickkids.on.ca/app), we selected only synonymous SNPs in the *CFTR* coding sequence (S1A Fig and S1 Table). The sSNPs were broadly examined for their influence on steady-state protein and mRNA levels in the CF bronchial epithelial cell line (CFBE41o^-^) and HeLa cells. The mutations G1584A, G2280A, T3339C, and A3870G frequently showed reduced mRNA levels in both HeLa and CFBE41o^-^ cells (Fig 1A), yet the total protein level demonstrated a cell-specific pattern and compared to wild-type CFTR was reduced mostly in HeLa cells, but not in CFBE41o^-^ cells (Fig 1B and S1B Fig). Interestingly, a cell-specific pattern of mRNA steady-state expression was observed for ΔF508 (Fig 1A), but the protein level remained equally low in both cell lines (Fig 1B). Of note, for the T2562G mutation, we detected unusual behavior. It significantly reduced the total protein expression (the sum of B and C bands) by 25%–30% in both HeLa and CFBE41o^-^ cells compared to that of wild-type CFTR (Fig 1B), while the mRNA level remained unchanged (Fig 1A). Like wild-type CFTR [25], T2562G-CFTR consisted of both core-glycosylated (immature, endoplasmic reticulum (ER)-resident, band B) and complex-glycosylated (mature, Golgi processed, band C) forms (S1B Fig). However, the ratio of C to B bands for T2562G-CFTR remained the same as that of wild-type CFTR (S1B Fig), implying a proportional reduction of both the mature form (band C) and the immature ER-resident form (band B). In well-differentiated human CF airway epithelia, T2562G-CFTR localized either at the apical membrane or intracellularly, whereas wild-type CFTR was localized only at the apical membrane and ΔF508-CFTR was retained intracellularly (Fig 1C).

Some sSNPs may alter CFTR mRNA splicing by changing the regulatory motifs of exonic splice enhancers [26]. Although we work exclusively with cDNA, which considers only full-length *CFTR* transcripts, we sought to address whether in the pre-mRNA of native *CFTR* the T2562G sSNP might alter splicing to reduce mRNA levels. Hence, we analyzed the effects of T2562G on mRNA splicing by using the minigene approach that was established to assess alternative splicing variants [27, 28]. The T2562G mutation is located in exon 15 of CFTR. Using highly sensitive on-chip capillary electrophoresis detection, which sensitively detects even background splicing in wild-type CFTR, we studied a minigene spanning exon 15 and parts of its flanking introns (S1C–S1E Fig). Of note, the T2562G sSNP did not induce any detectable alternative splicing product above the background of wild-type CFTR (S1C–S1E Fig). This result is consistent with previous observations, which report no changes in the mRNA splicing pattern of CF patients [29], and alternatively spliced products are only detected when T2562G occurs with other (silent) mutations [26, 28].

### The T2562G mutation alters CFTR structure

The reduced protein expression and altered localization of T2562G-CFTR when compared to wild-type CFTR raised the intriguing possibility that T2562G might trigger an alternative channel conformation, which renders T2562G-CFTR a better client for quality control machinery. We therefore investigated the degradation of T2562G-CFTR protein by the proteasome. T2562G-CFTR showed modest, but significantly enhanced ubiquitination and higher susceptibility to proteasomal degradation compared to wild-type CFTR (S2A–S2D Fig). Two E3-ubiquitin–ligating enzymes, the cytosolic C-terminus of Hsc70-interacting protein (CHIP) and the ER-membrane–bound RING domain protein RMA1 (also known as RING Finger Protein 5 [RNF5]), participate in the surveillance of CFTR biogenesis [30]. While RMA1 recognizes folding defects during or soon after translation of both wild-type and ΔF508-CFTR, CHIP inspects their folding status at a later time point, at least after synthesis of nucleotide-binding domain 2 (NBD2) [31]. RMA1 and CHIP both displayed increased binding to T2562G-CFTR than to wild-type CFTR (S2E–S2G Fig), corroborating the observed reduction of the amount of T2562G-CFTR protein (Fig 1B). Using pulse-chase experiments in HeLa cells, we compared the maturation efficiency of wild-type and T2562G-CFTR by monitoring the conversion of newly synthesized immature core-glycosylated band B to mature complex-glycosylated band C. The conversion of the band B to band C was not significantly different between T2562G-CFTR and wild-type CFTR (S2H and S2I Fig). To test whether lower steady-state protein levels of T2562G-CFTR (Fig 1B) might result also from plasma membrane instability, we measured the stability of membrane-localized CFTR by using biotinylation of nonpermeabilized cells [30]. The stability of plasma membrane–localized wild-type and T2562G-CFTR was comparable at early time points (S2J Fig). However, some enhancement of T2562G-CFTR plasma membrane stability was apparent at 24 h (S2J Fig), which might, in part, offset its proteasomal degradation.

**Fig 2:**
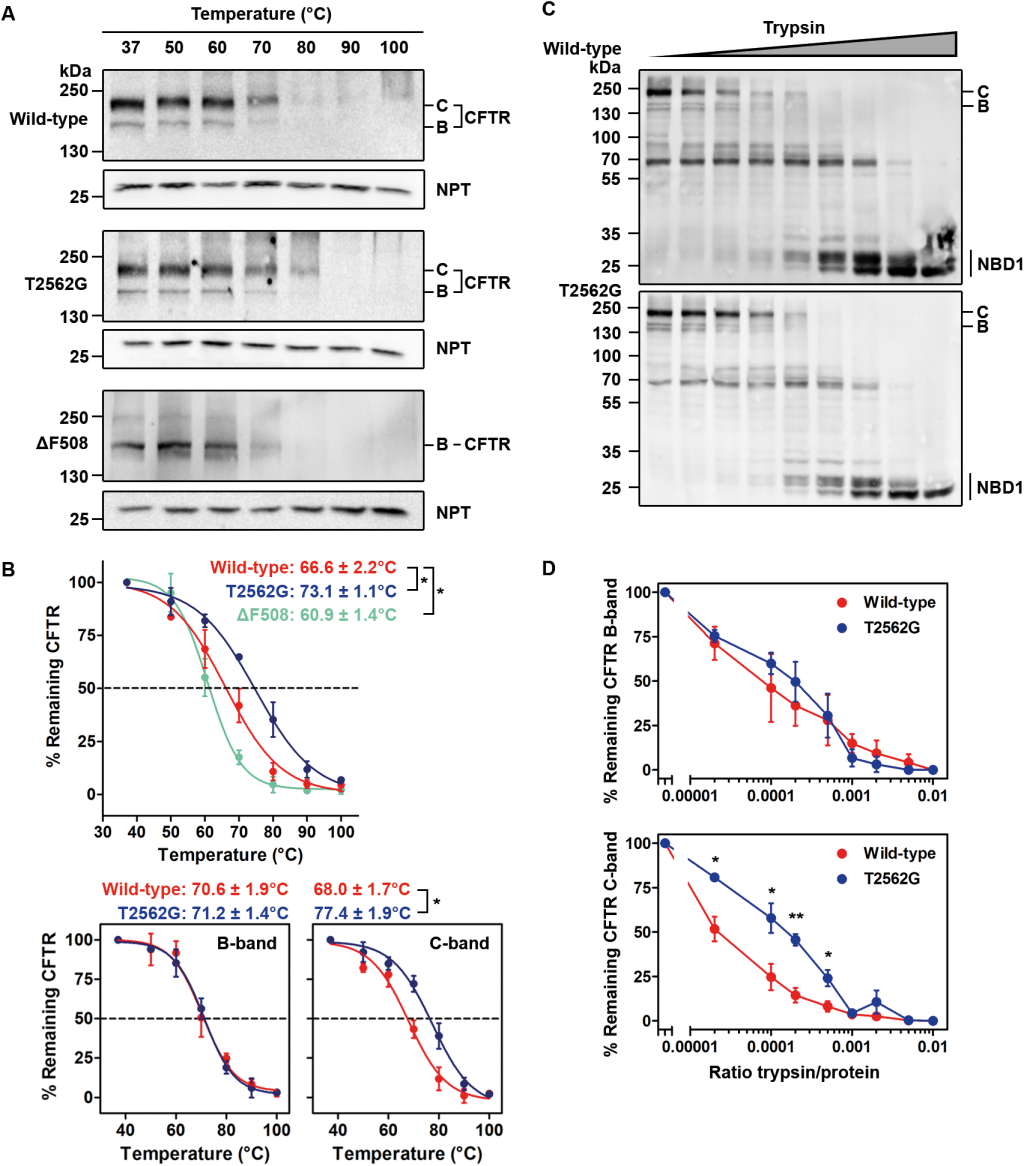
The T2562G synonymous single nucleotide polymorphism (sSNP) alters CFTR stability. (**A**) Representative immunoblots or western blots (WB) showing temperature-induced aggregation of CFTR variants. B and C denote immature and mature CFTR protein, respectively. (**B**) Quantification of the thermal aggregation propensity for the CFTR variants from panel A. The total amount of CFTR protein (sum of bands B and C, upper panel) or band B and band C separately, respectively, (bottom panel) at the indicated temperatures relative to those at 37°C is shown. Thermal aggregation temperatures (*T*_*a*_) are means ± SEM (*n* = 4–7); * *P* < 0.05. (**C**) Representative WBs (*n* = 4) of limited trypsin digestion of wild-type and T2562G-CFTR in semi-intact HeLa cells probed with anti-CFTR nucleotide-binding domain 1 (NBD1) (660) antibody. NBD1 indicates NBD1-containing fragments; numbers on the left are molecular mass standards. (**D**) Quantification of bands B and C of CFTR from panel C relative to untreated samples (set to 100%). Data are means ± SEM (*n* = 3); * *P* < 0.05, ** *P* < 0.01. The underlying data of panels B and D can be found in S1 Data.

Together, the enhanced ubiquitination and binding of T2562G-CFTR to CHIP and RMA1, albeit modest in magnitude, suggest that T2562G induces subtle alterations in CFTR structure that are detected by quality-control machinery. These slight changes escape detection in kinetic experiments (i.e., pulse-chase assays). However, over time, these small differences in CFTR structure and cell-surface stability might accumulate and be detected in steady-state experiments. Thus, to further evaluate structural rearrangements in T2562G-CFTR, we used limited proteolysis and thermal aggregation assays [32]. Lysates of cells expressing CFTR variants were exposed to different temperatures and the fraction of membrane-bound, nonaggregated CFTR was determined (Fig 2A and 2B). As a measure of the thermal aggregation propensity of the CFTR protein, the aggregation temperature (*T*_*a*_) was defined as the temperature at which 50% of CFTR protein remained membrane-soluble [32]. T2562G-CFTR displayed a higher *T*_*a*_ (73.1 ± 1.1°C; mean ± SEM) than wild-type CFTR (66.6 ± 2.2°C) (Fig 2A and 2B). Interestingly, the magnitude of thermal stabilization achieved by the T2562G mutation (~6°C) was equivalent to the destabilizing effect of the ΔF508 mutation (*T*_*a*_ = 60.9 ± 1.4°C) (Fig 2A and 2B). When compared with wild-type CFTR, T2562G-CFTR reproducibly displayed subtle structural differences: the T2562G sSNP decreased slightly the susceptibility of CFTR to limited proteolysis (Fig 2C and 2D and S3 Fig). Of note, the similarity of the proteolytic patterns of wild-type and T2562G-CFTR (Fig 2C and S3A Fig) argues against large structural rearrangements and instead suggests that the T2562G sSNP causes local conformational changes. Consistent with this idea, we detected no discernible differences in the proteolytic susceptibility and thermal stability of band B, only of band C (Fig 2B, bottom panel and Fig 2D). This suggests that the effects of T2562G are on the overall topology of CFTR.

### The T2562G mutation alters CFTR single-channel function

To address the impact of the T2562G sSNP on CFTR function as a regulated Cl^-^ channel, we studied individual CFTR Cl^-^ channels in excised inside-out membrane patches with the patch-clamp technique. For 2 reasons, we did not study macroscopic CFTR Cl^-^ currents to learn how the T2562G sSNP influences CFTR function: First, studies of macroscopic CFTR Cl^-^ currents do not distinguish between mutation effects on CFTR expression (i.e., channel number) and CFTR function (i.e., conductance and gating). Second, we reasoned that the effects of an sSNP on CFTR function would likely be slight and therefore not easily resolved when studying macroscopic CFTR Cl^-^ currents. We anticipated that high-resolution single-channel recording would be required to discern the impact of the T2562G sSNP on CFTR function.

**Fig 3:**
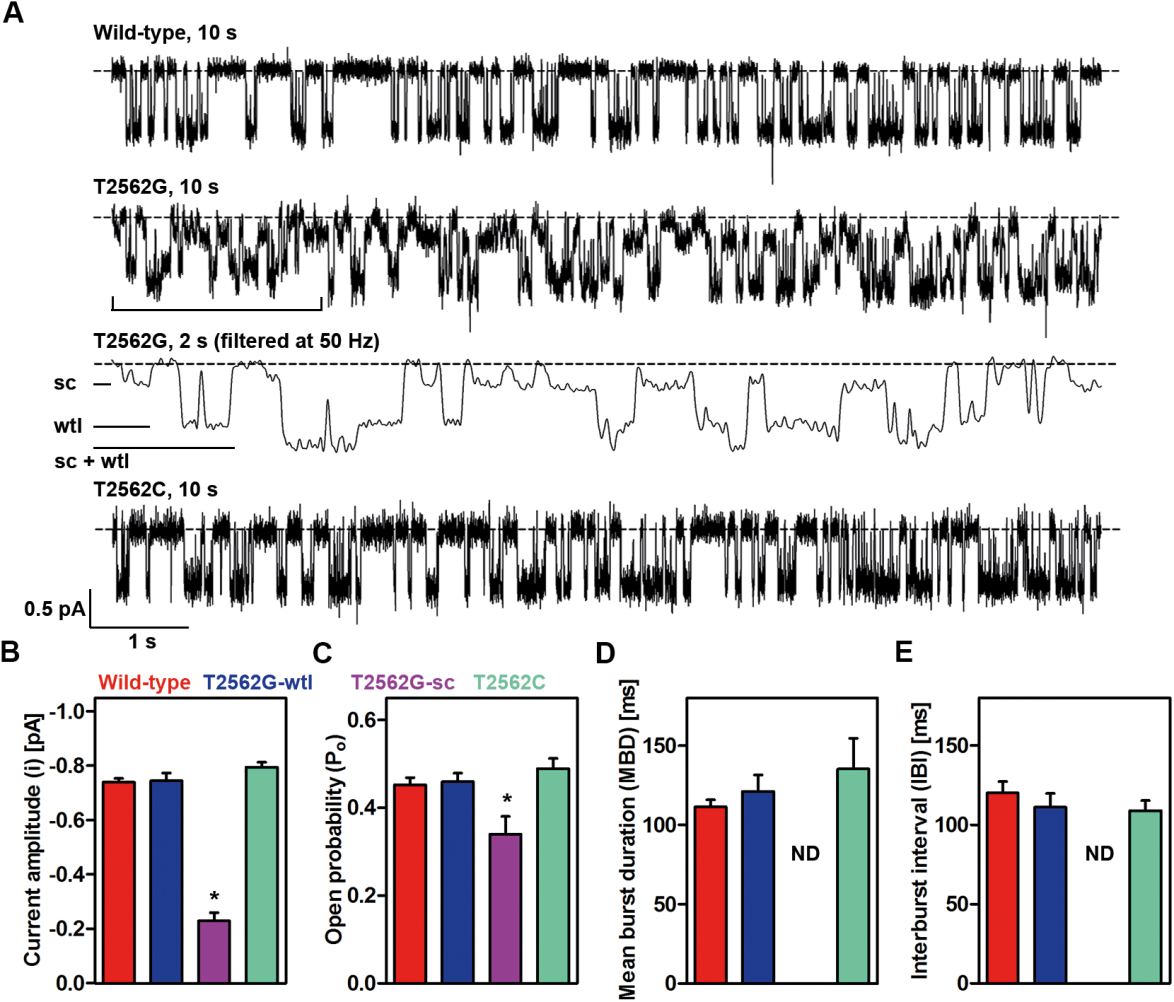
T2562G-CFTR exhibits 2 types of channel openings. (**A**) Representative single-channel recordings of CFTR variants in excised inside-out membrane patches from CHO cells. For wild-type and T2562C-CFTR, the membrane patches contained 1 channel; for T2562G-CFTR, it contained 2 channels—one with small conductance (sc) and the other with wild-type–like (wtl) openings. Dashed lines indicate the closed channel state and downward deflections correspond to channel openings. For T2562G-CFTR, the indicated 2-s portion is shown on an expanded time scale after filtering digitally. (**B–E**) Single-channel current amplitude (*i*), open probability (*P*_o_), mean burst duration (MBD), and interburst interval (IBI) of CFTR variants. Data are means ± SEM (*n* = 5–9); * *P* < 0.05 versus wild-type CFTR; ND, not determined. The underlying data of panels B–E can be found in S1 Data.

Once phosphorylated by protein kinase A (PKA), wild-type human CFTR forms a low-conductance, Cl-selective channel regulated by cycles of ATP binding and hydrolysis [22, 33]. In single-channel recordings, wild-type CFTR exhibits a bursting pattern of channel gating with channel openings interrupted by brief, flickery closures, separated by longer closures between bursts (Fig 3A). Strikingly, 2 populations of T2562G-CFTR channels were distinguished following PKA-dependent phosphorylation (Fig 3A, S4 Fig and S2 Table). The first population of T2562G-CFTR channels (wild-type–like [wtl] population) exhibited characteristics identical to those of wild-type CFTR (Fig 3 and S5 Fig). Wild-type–like openings of T2562G-CFTR had the same current amplitude and conductance as wild-type CFTR (Fig 3B and S5 Fig), demonstrating that the channel pore was unaltered from wild-type CFTR. Similarly, wtl openings of T2562G-CFTR exhibited a gating pattern with an identical mean burst duration (MBD) (the average duration of channel openings), interburst interval (IBI) (the average duration of long channel closures) and open probability (*P*_o_) (a measure of single-channel activity) as wild-type CFTR (Fig 3C–3E). These data argue that the regulation of wtl openings of T2562G-CFTR by intracellular ATP is the same as wild-type CFTR. By contrast, the second population of T2562G-CFTR channels (small-conductance [sc] population) was distinguished by the small size of channel openings, with a single-channel conductance about half that of wild-type CFTR and a small, but significant reduction in *P*_o_ (Fig 3 and S5 Fig). We interpret these results to suggest that the sc population of T2562G-CFTR Cl^-^ channels has a constricted pore for Cl^-^ flow and ATP is slightly less efficacious in stimulating channel gating. Of note, sc openings of wild-type CFTR are very rare events and they are distinct from those of T2562G-CFTR (S2 Table); they are also different to the brief sojourns to subconductance states observed with wild-type human CFTR [34].

Several lines of evidence suggest that the sc and wtl channels of T2562G-CFTR are independent channel populations. First, both wtl and sc T2562G-CFTR channels were observed together (Fig 3A, S4–S6 Figs and S2 Table) or by themselves (S4B and S4C Fig and S2 Table). Second, the occurrence of membrane patches with only 1 type of active channel or with dissimilar numbers of wtl and sc channels argues against the occurrence of multiple conductance states [35], mode switching [36], or coupled transitions [37] (S4B and S4C Fig and S2 Table). Third, binomial analysis of membrane patches with 1 sc and 1 wtl channel demonstrated that experimentally measured values of the 3 conductance levels—*P*_(*0*)_, *P*_(*1*)_, and *P*_(*2*)_—differed from their predicted values (S6 Fig), implying that sc and wtl channels are either dependent on each other or are nonidentical channels. To distinguish between these possibilities, we measured the cooperativity ratio (CR) [38]. In membrane patches with 1 sc and 1 wtl channel, the CR was 2 ± 0.1 (*n* = 3), implying that sc and wtl channels are independent, nonidentical channels. Thus, the subtle structural changes caused by the T2562G sSNP, which increase CFTR stability, are detrimental to channel function.

### The effects of the T2562G sSNP are tRNA-dependent and tissue-specific

**Fig 4:**
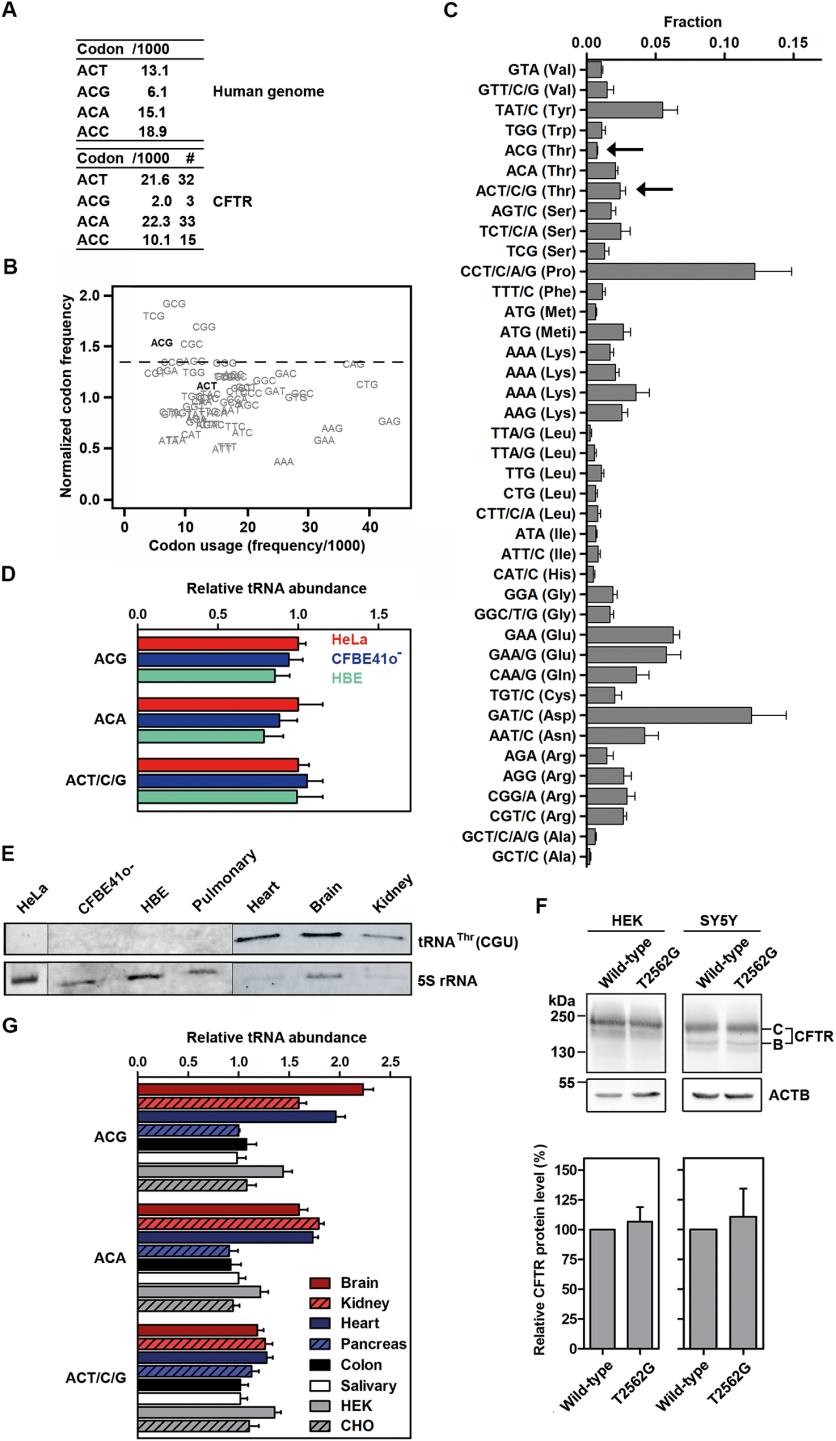
tRNA^Thr^(CGU) level is very low in CFBE41o^-^, primary human bronchial epithelial (HBE) cells, and pulmonary tissues. (**A**) Frequency of the Thr codons per thousand (/1,000) and as absolute numbers (#). (**B**) Ribosome dwelling occupancy at the ACG codon is highest in CFBE41o^-^ cells. The graph shows the relationship between dwelling occupancy (normalized codon frequency), as determined by ribosome profiling, and genome codon usage. Two Thr codons, ACG and ACT, are highlighted in black. The dashed line denotes the upper boundary (90% confidence interval) for ribosome dwelling occupancy. (**C**) Microarray analysis of the absolute tRNA concentration in HeLa cells. Data are means ± SEM (*n* = 3). tRNA probes are depicted with their cognate codon and the corresponding amino acid; Meti, initiator tRNA^Met^. Arrows denote the 2 tRNAs decoding ACG and ACT codons. (**D**) Comparative microarrays of the abundance of tRNAs^Thr^ in CFBE41o^-^ and 4 cystic fibrosis (CF) patient–derived HBE cells relative to HeLa tRNAs for which the absolute concentration of tRNA^Thr^ is determined in C. Data are means ± SEM (*n* = 4–5). For the full comparison of the complete tRNA set, see S8 Fig. (**E**) Northern blot of total tRNA isolated from different cells and human tissues (pulmonary, heart, brain, and kidney) probed with fluorescently labeled tRNA^Thr^(CGU) and 5S rRNA probes. HBE denotes primary HBE cells from ΔF508 homozygous CF patients and Pulmonary indicates pulmonary tissue from a non-CF individual. (**F**) Steady-state CFTR expression in kidney (HEK 293) and neuronal (SH-SY5Y) cell lines. Total expression (i.e., the sum of bands B and C) was normalized to that of NPT and ACTB and was related to wild-type CFTR, which was set to 100%. Data are means ± SEM (*n* = 3). (**G**) Comparative microarrays of the abundance of tRNAs^Thr^ in various tissues and cell lines compared to HeLa tRNAs, which are set as 1 using the same format as in D. The underlying data of panels B–D and F–G can be found in S1 Data.

How might a synonymous mutation alter protein conformation and function? The T2562G sSNP exchanges the Thr854-ACT codon for a Thr854-ACG triplet (i.e., exchanging a T for G nucleotide) (Fig 4A). A/T nucleotides have lower propensity to partition in secondary interactions than G/C nucleotides. The unaltered mRNA levels of the T2562G-CFTR (Fig 1A) argue against any significant global changes in the mRNA secondary structure and stability through the T2562G sSNP; nevertheless, subtle local changes might escape detection. Thus, we also exchanged the Thr854ACT triplet for its 2 other synonymous alternatives, ACC and ACA (Fig 4A). Both T2562C- and T2562A-CFTR were indistinguishable from wild-type CFTR in their mRNA and protein expression levels, thermal stability (S7 Fig), and channel function (Fig 3 and S5 Fig), suggesting that the observed effect of the T2562G sSNP is specific for the ACG codon.

All 4 Thr codons are of moderate genome usage, differing only by 2–3-fold (Fig 4A), while the difference between high- and low-abundance codons is 7.5–8.7-fold (S3 Table). Next, we used ribosome profiling to assess the relative average speed of translation of each Thr codon in CFBE41o^-^ cells. The residence frequency of a codon in the ribosomal A site (that is, the site accepting the aminoacyl-tRNA) correlates with the ribosome dwell time at that particular codon and is proportional to the codon’s translational speed [21, 39]. We calibrated the ribosome-protected fragments on the ribosomal A site by using the 5′ ends of the sequencing reads as previously described [40]. Strikingly, the ACG codon was among the codons with the highest ribosomal occupancy, implying that it is among the most slowly translated codons in CFBE41o^-^ cells (Fig 4B). By contrast, the ACT codon less frequently dwelt in the ribosomal A site and was translated with much higher velocity (Fig 4B).

The concentration of the cognate tRNA is one major determinant of ribosome speed at a codon. To address whether ribosome occupancy at Thr codons correlates with the concentration of their cognate tRNAs, we determined the absolute tRNA concentration in HeLa cells (Fig 4C) and related the abundance of each single tRNA to that of CFBE41o^-^ cells and CF patient–derived primary human bronchial epithelial cells (HBEs) by using tRNA-tailored microarrays (Fig 4D, S8A Fig and S1 Text). tRNA isoacceptors (that are different tRNA species carrying the same amino acids, but with different anticodons) varied greatly in their concentrations, spanning up to an order of magnitude (Fig 4C). For example, compare tRNA^Pro^ that pairs to Pro (CCT/C/A/G) codons and tRNA^Thr^ that reads ACG codon (Fig 4C). While tRNA^Thr^(UGU) that pairs to ACT codons is among the moderately abundant tRNAs, tRNA^Thr^(CGU) decoding the ACG codon is one of the rarest tRNAs in HeLa (Fig 4C, both designated with arrows). Comparative microarray analysis demonstrated that the levels of the tRNA^Thr^(CGU) decoding the ACG codon were similar (and hence, equally rare) in CFBE41o^-^ and 4 CF patient–derived primary HBE cells (Fig 4D and S8D Fig). To verify this result, we performed northern blotting using specific probes that spanned the anticodon loop tRNA^Thr^(CGU). No tRNA^Thr^(CGU) signal was detected in CFBE41o^-^ cells, CF patient–derived primary HBE cells, or pulmonary tissue from a non-CF individual, implying very low abundance, below the detection limit of the northern blot (Fig 4E). Of note, the higher ribosomal occupancy of approximately 50% at the ACG codons compared to the ACT codons (Fig 4B) mirrors the concentration difference between their cognate tRNAs (Fig 4C and 4D; see also S1 Text). Thus, the slower translation of ACG compared to ACT detected by ribosome profiling (Fig 4B) correlates with the rare abundance of its cognate tRNA^Thr^(CGU).

Unexpectedly, we measured far higher concentrations of tRNA^Thr^(CGU) in other human tissues, including the heart, brain, and kidney (Fig 4E). In 2 laboratory cell lines of kidney (HEK 293T) and neuronal (SH-SY5Y) origin, T2562G-CFTR expression did not differ from that of wild-type CFTR (Fig 4F), suggesting that when the cellular level of tRNA^Thr^(CGU) is high (Fig 4A), the effect of T2562G sSNP remains dormant. Of note, the ACG codon is rarely used in CFTR to encode Thr (Fig 4A). Strikingly, among tissues that naturally express CFTR, including the pancreas, colon, and salivary glands, the amounts of all tRNAs^Thr^ are equal (Fig 4G), with tRNA^Thr^(CGU) being the rarest among the 3 tRNA^Thr^ isoacceptors (for further information, see S1 Text). We conclude that the rare usage of the ACG codon in CFTR (Fig 4A) correlates with the very low abundance of the cognate tRNA^Thr^(CGU) in tissues naturally expressing CFTR. The mutated ACG codon is read by a rare tRNA in the cell lines used in this study (CFBE41o^-^, CHO, and HeLa cells; Fig 4D, 4E and 4G). However, the levels of other tRNA isoacceptors also differ between CFBE41o^-^ and HeLa cells (S8D Fig). As a result, the effects of other sSNP involving those differing tRNA isoacceptors might vary between CFBE41o^-^ and HeLa.

Finally, we compared the tRNA concentration in both HeLa and CFBE41o^-^ cells with genomic codon usage. The tRNA concentration in both cells correlated poorly with genomic codon usage (S8B and S8C Fig), suggesting that codon usage is a poor predictor of the speed of translation of each single codon. Taken together, variation in the amount of tRNA^Thr^(CGU) measured in different human tissues argues that despite common codon usage, tRNAs modulate the effects of sSNP in a tissue-specific manner.

### Enhanced tRNA^Thr^(CGU) levels rescue the effects of the T2562G mutation

**Fig 5:**
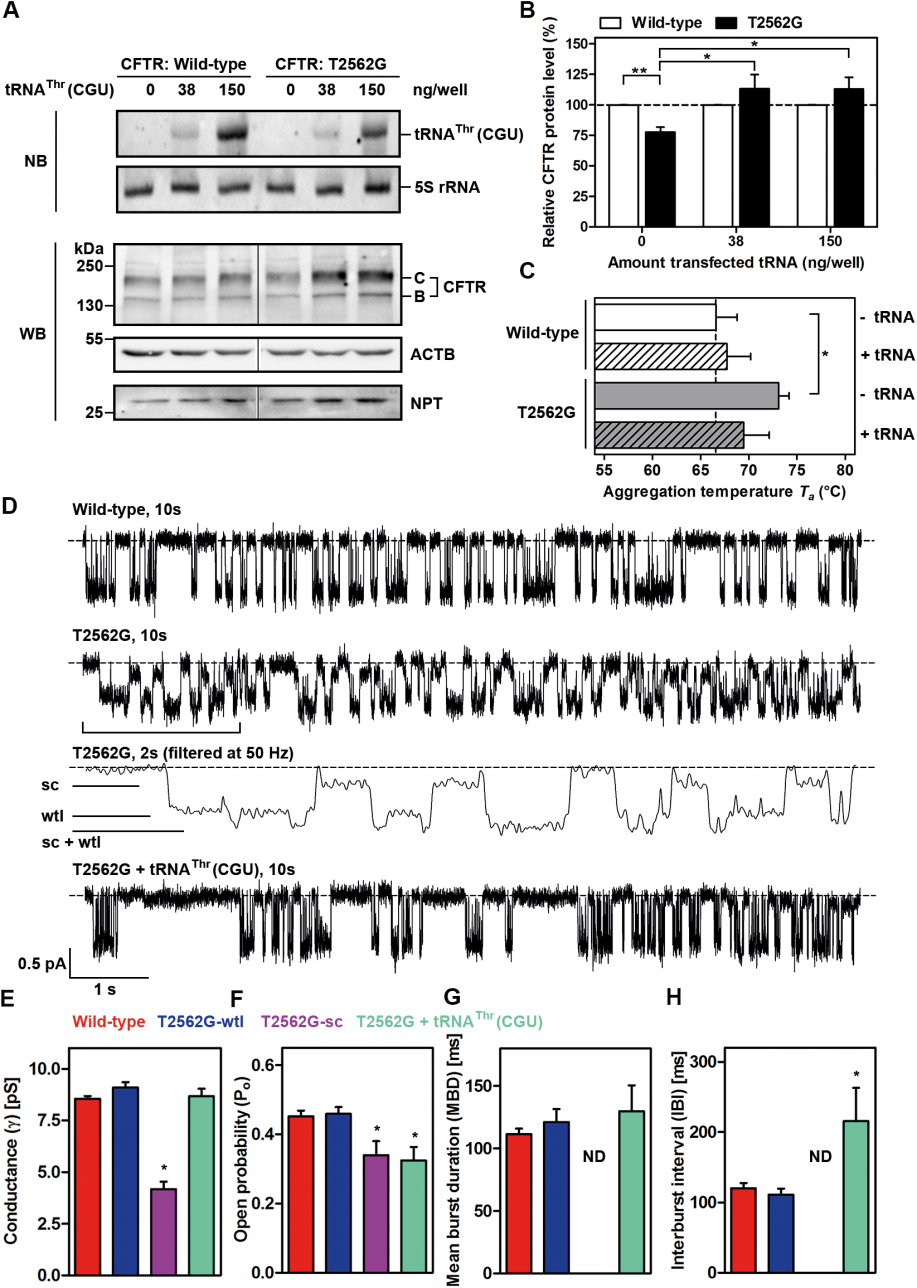
Increased tRNA^Thr^(CGU) levels ameliorate the expression and stability defects and abolish small-conductance openings of T2562G-CFTR. (**A**) Representative northern blot (NB) and WB analysis of HeLa cells cotransfected with tRNA^Thr^(CGU) and either wild-type or T2562G-CFTR. (**B**) Quantification of T2562G-CFTR from A (details in Fig 1B) and presented relative to those of wild-type CFTR (set to 100%). Values are means ± SEM (*n* = 5–7); * *P* < 0.05; ** *P* < 0.01. (**C**) Thermal aggregation propensity assay upon cotransfection with 150 ng tRNA^Thr^(CGU) (+tRNA) compared to untransfected controls (-tRNA). Values are means ± SEM (*n* = 3–5); * *P* < 0.05 versus wild-type CFTR. (**D**) Representative single-channel recordings of wild-type, T2562G-, and T2562G-CFTR cotransfected with tRNA (T2562G + tRNA^Thr^(CGU)) in excised inside-out membrane patches from CHO cells. The dashed lines indicate where channels are closed and downward deflections of the traces correspond to channel openings. For T2562G-CFTR, the indicated 2-s portion is shown on an expanded time scale after filtering at 50 Hz. For wild-type and T2562G-CFTR coexpressing tRNA^Thr^(CGU), membrane patches contained 1 active channel; for T2562G-CFTR, it contained 2 channels—one with sc openings and the other with wtl. (**E–H**) Single-channel conductance (γ), open probability (*P*_o_), mean burst duration (MBD), and interburst interval (IBI) of the indicated CFTR variants. Data are means ± SEM (*n* = 6–10); * *P* < 0.05 versus wild-type CFTR. In E–H, control data are the same as Fig 3C–3E and S5C Fig. The underlying data of panels B–C and E–H can be found in S1 Data.

If the aberrant conformation and function of T2562G-CFTR is indeed a consequence of delayed translation at this sSNP by the low concentration of the cognate tRNA^Thr^(CGU), then the phenotype should be rescued by increasing the speed of translation at this codon through elevation of cellular tRNA^Thr^(CGU) levels. To test this idea, we transiently cotransfected T2562G-CFTR and in vitro synthesized, uncharged tRNA^Thr^(CGU). Elevated levels of the rare tRNA^Thr^(CGU) caused a tRNA concentration–dependent increase in T2562G-CFTR expression (Fig 5A and 5B and S9A Fig) and restored the aggregation temperature of T2562G-CFTR to that of wild-type CFTR (Fig 5C). Transfection of tRNA was without effect on translation (S9A–S9C Fig), arguing against global alteration of translation. Moreover, the transfected tRNA^Thr^(CGU) was translationally active, as it was present also in the polysomal fraction (S9D Fig). Conversely, down-regulation of tRNA^Thr^(CGU) using shRNA treatment noticeably decreased the T2562G-CFTR steady-state protein level (S9E and S9F Fig) with only marginal effects on global translation (S9G Fig).

Finally, we investigated whether accelerating ribosomal passage at the sSNP with tRNA^Thr^(CGU) also ameliorated its functional defects. Elevating the tRNA^Thr^(CGU) concentration rescued the conductance defect of the sc population of T2562G-CFTR channels (Fig 5D–5H), albeit without altering its minor gating defect. Only large-amplitude openings of T2562G-CFTR were observed, with conductance similar to that of wild-type CFTR (Fig 5D–5H and S2 Table). We conclude that the effects of the T2562G sSNP are clearly tRNA-dependent, and increasing the cellular tRNA^Thr^(CGU) concentration rescued both the expression and single-channel conduction defects of T2562G-CFTR.

## Discussion

**Fig 6:**
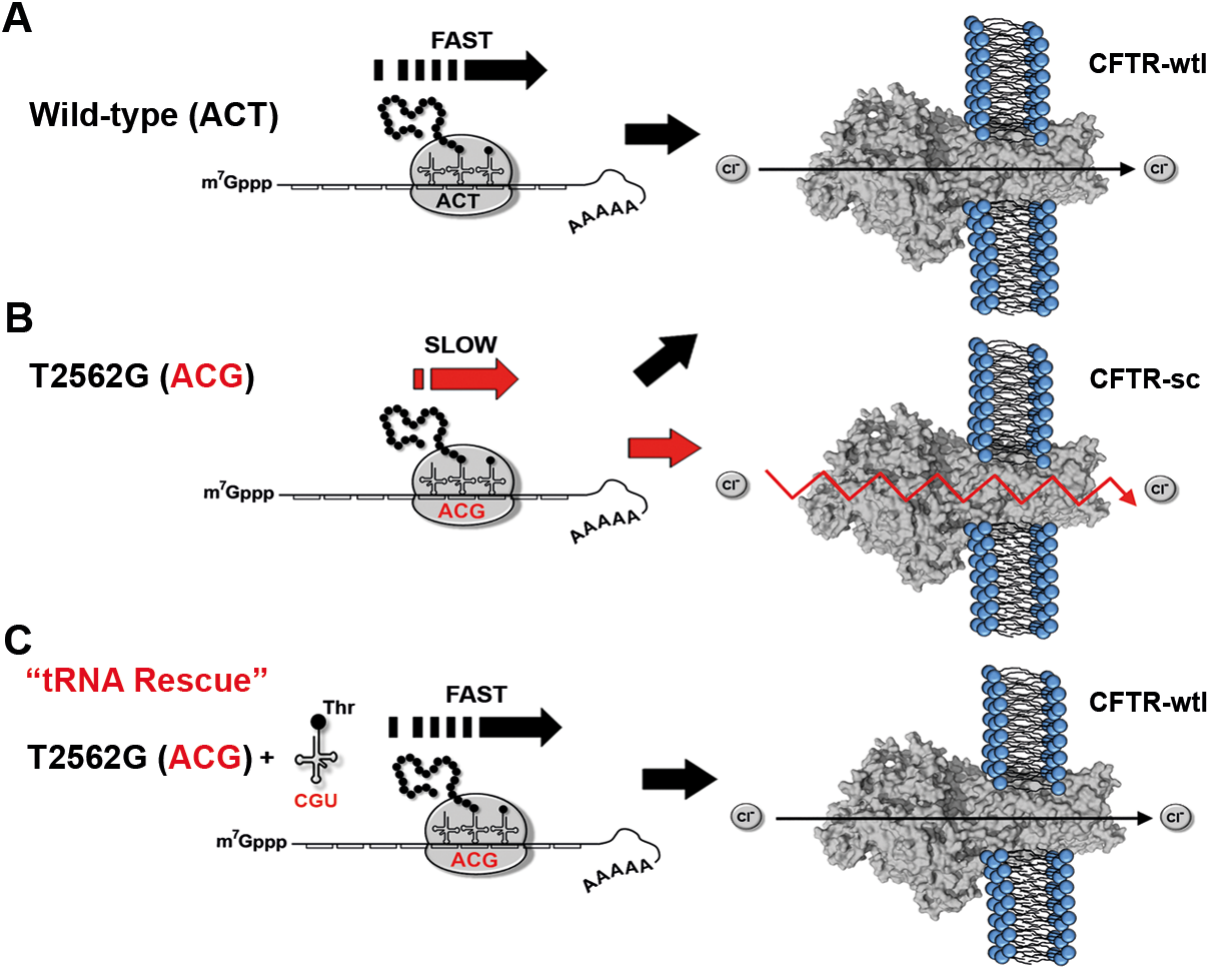
The synonymous single nucleotide polymorphism (sSNP) T2562G inverts local translation speed in CFTR mRNA, which can be rescued by tRNA^Thr^(CGU). (**A**) Thr-854–encoding codon ACT in wild-type CFTR is translated fast, as its cognate tRNA^Thr^(AGU) is relatively abundant. (**B**) T2562G sSNP converts the ACT triplet to ACG codon, which is read by the rare cognate tRNA^Thr^(CGU) and reduces local ribosomal speed. Stochasticity in the delivery of tRNA^Thr^(CGU) cognate to the ACG codon creates variations in the intimate translation speed of each ACG codon at this position and hence generates 2 distinct CFTR channel populations, one with wild-type–like (wtl) CFTR properties and a second with a reduced conductance and a more compact structure (small-conductance [sc] population). (**C**) Increase of the cellular level of tRNA^Thr^(CGU) pairing to the mutated ACG codon restores ribosome speed at the rare Thr-ACG codon and rescues the CFTR conductance defect.

Our results demonstrate that the T2562G sSNP induces local changes in translation velocity, giving rise to more stable channels with a greatly reduced single-channel conductance (Fig 6A and 6B). The effect is clearly tRNA-dependent, as increasing the cellular concentration of tRNA cognate to the mutant codon rescues the stability and conductance defects of T2562G-CFTR (Fig 6C).

Translation rate is maximized at codons read by highly abundant tRNAs and minimized at codons with rare tRNAs. Thereby, the selection of codons translated at different speeds is not random [3] and shapes the kinetics of mRNA translation to regulate cotranslational protein folding [13, 41–43]. Consistent with this idea, substitution with slow-translating codons in fast-translating regions (and vice versa) might be incompatible with cotranslational protein folding and deleterious for protein integrity. Our results suggest that the T2562G sSNP–induced CFTR dysfunction is through inversion (i.e., slowing down) of local ribosomal speed at the mutant ACG codon. Of note, these data provide the first direct evidence for the central role of tRNA in mediating the effects of an sSNP. The T2562G sSNP exchanges the ACT codon for the ACG triplet. Despite the similar usage of ACT and ACG, which differ only by 2–3-fold (Fig 4A), the ACG triplet has a measurable effect on translation kinetics. tRNA^Thr^(CGU) cognate to the ACG triplet is present at very low abundance in human bronchial epithelial cells, rendering the ACG codon very slowly translated in these cells. tRNAs of the same kind are not uniformly distributed within the cell, which creates inherent variation in the speed of translation of a codon. We reason that such local differences in the tRNA concentration and in general the stochastic nature of tRNA delivery to the ribosome result in production of 2 populations of T2562G-CFTR channels: one identical to wild-type (wtl) and a second with reduced (sc) single-channel conductance (Fig 6B). tRNA^Thr^(CGU) is far more abundant in other tissues. Hence, the ACG codon will be translated with different velocities in diverse tissues, suggesting a tissue-specific effect of the T2562G sSNP, which for CFTR might be restricted only to epithelial cells.

Strikingly, codon usage within the CFTR sequence deviates greatly from human genomic codon usage, with the ACG codon being rarely used in CFTR to encode Thr (Fig 4A). The very low concentration of the cognate tRNA^Thr^(CGU) in bronchial epithelial cells and tissues, which are a key site of CFTR expression, suggests that the ACG codon might have been under selection pressure. Consistent with this idea, wild-type CFTR has in total 3 ACG codons, all of which are located in the context of fast-translating codons pairing to highly abundant tRNAs. Thus, most likely the effects of these 3 naturally occurring ACG codons in wild-type CFTR remain dormant. By contrast, the T2562G sSNP introduces a slowly translating ACG codon in a region enriched with other slowly translating codons, emphasizing the importance of sequence context for the effects of an sSNP. It should be noted that sSNPs have diverse effects that are not always tRNA-dependent. For example, the sSNP at Ile507 of CFTR, which accompanies the ΔF508 mutation, stabilizes secondary mRNA structure in the vicinity of the mutation and most likely contributes to ΔF508-CFTR misfolding by decreasing the mRNA translation rate [44].

Folding of CFTR is cotranslational [45], guided by intensive interactions with cytosolic and ER-resident chaperones [30] and most likely orchestrated by translation kinetics [46]. Importantly, the structural coupling of independently folded CFTR domains and extensive domain–domain interface contacts maintain channel stability and function [47–50]. The ΔF508 mutation, which causes a dramatic reduction of functional plasma membrane–resident CFTR, has minimal impact on the protein backbone and folding of NBD1, but greatly compromises interdomain interfaces [47, 50]. Since the T2562G mutation causes no discernible effects on the core glycosylated form (band B), this suggests that T2562G-CFTR folds close to its native conformation. The larger effect on stability and function of the complex glycosylated form (band C) suggests that the T2562G sSNP likely affects the conformational dynamics of CFTR. The atomic structure of zebrafish (*Danio rerio*) CFTR identifies an N-terminal ‘lasso’ motif, which wraps around the central axis of the protein and likely regulates channel gating through interactions with the R domain [51, 52]. Although it is not possible to identify Thr854-interacting partners in the atomic structure of zebrafish CFTR [52], it is conceivable that Thr854 might interact with the lasso motif and, hence, that the T2562G sSNP might disrupt this interaction. Thr854 is located at the edge of the R domain, close to membrane-spanning domain 2 (MSD2), in a structured α-helical segment (S1A Fig); thus, it is positioned at the interface between the lasso motif and MSD2 [52]. T2562G-induced changes in Thr854 codon speed might therefore alter this α-helix, destroying interface interactions, leading to changes in CFTR conductance. Alternatively, the T2562G sSNP might impact interdomain interactions of the R domain [53] with other domains of CFTR to influence channel conductance. A further possibility is that the T2562G mutation alters R domain phosphorylation, leading to reduced CFTR expression [54]. Clearly, despite the local subtle effects of the T2562G sSNP, which are only in the vicinity of the mutation, the T2562G sSNP appears to stabilize the final 3D topology of CFTR, most likely by influencing R domain–mediated interdomain interactions, which reduce conformational dynamics and perturb channel conductance.

CFTR is one of the most polyvariant human genes and it is now recognized that CFTR mutant alleles frequently carry combinations of mutations [23, 55]. T2562G is one of the most common SNPs in the *CFTR* gene [56, 57] with a prevalence of 34% in the general population. Although this sSNP, by itself, does not cause CF, it is prevalent in patients with CFTR-related disorders [26, 58–60], which argues that sSNPs have the potential to epistatically modulate the effects of disease-causing mutations, thereby modifying disease severity. Building on this idea, the Ile507 sSNP along with the ΔF508-mutation alters the response of ΔF508-CFTR to small molecule CFTR correctors [61], implying the potential contribution of epistatic mutations to personalized medicine.

Translation kinetics is an integral feature of CFTR folding and tunes synthesis at critical nodes of the CFTR cotranslational folding landscape [46]. Consistent with this idea, 2 studies show that modulation of translation velocity is a robust strategy to correct folding errors in CF mutants [62, 63]: First, a global decrease of translation kinetics by varying codon choice [62] and second, knockdown of a ribosomal protein greatly increases the folding efficiency of ΔF508-CFTR [63]. In conclusion, our results demonstrate that the complex effects of the T2562G sSNP on CFTR function are most likely a result of altered local kinetics of mRNA translation. We speculate that the inversion of codon speed induced by the T2562G sSNP is applicable to other proteins in a tissue-specific fashion. SNPs introduce variability into an individual’s genome composition, which might influence disease risk, the spectrum of disease symptoms, and ultimately, therapeutic response.

## Materials and methods

### Ethics statement

Pulmonary tissues from lung biopsies were provided by Dr. Raymond Frizzell and the Health Sciences Tissue Bank (HSTB). HSTB is covered by the University of Pittsburgh IRB approval #0506140.

### Cell culture, tissues, and transfection

HeLa cells (American Type Culture Collection [ATCC] no. CRM-CCL-2), HEK 293 cells (ATCC no. CRL-1573), SH-SY5Y (German cell collection (DSMZ no. ACC209) and immortalized CFBE41o^-^ cells (kind gifts of Karl Kunzelmann, University of Regensburg, Germany; Eric Sorscher, University of Alabama, United States; and Dieter Gruenert, University of California San Francisco, US) were maintained in Dulbecco's modified Eagle's medium (DMEM; PAN Biotech) or Earle's minimal essential medium (MEM; Biochrom), supplemented with 10% fetal calf serum (FCS; PAN Biotech) and 2 mM L-glutamine (Gibco). CHO cells (ATCC no. CCL-61) were grown in Ham’s F-12 nutrient medium supplemented with 10% fetal calf serum (both from Life Technologies). For patch-clamp experiments, HeLa or CHO cells were seeded onto glass coverslips 24 h before transfection. All cells were incubated at 37°C in a humidified atmosphere with 5% CO_2_. CFBE41o^-^ cells were cultivated on collagen- and fibronectin-coated cell culture dishes. All cells were frequently checked for mycoplasma contamination (Venor*GeM* mycoplasma detection kit; Biochrom).

The cDNAs of wild-type CFTR, ΔF508-CFTR and CFTR sSNP variants were subcloned into the pcDNA3 vector (Life Technologies) and transfected by using either Lipofectamine LTX, Lipofectamine Plus (Life Technologies), or by using polyethylenimine (PEI, linear [25,000], Polysciences). Constructs were verified by sequencing. In patch-clamp experiments, CFTR cDNAs were cotransfected with those of GFP and then 36–60 h later, GFP-expressing cells were selected for study [64].

Human tRNA sequences were extracted from the Genomic tRNA Database (http://gtrnadb.ucsc.edu). Single-stranded DNA oligonucleotides resembling full-length tRNAs were annealed to obtain double stranded, full-length tDNA templates flanked at the 5′-end with T7 promoter sequence and at the 3′-terminus with CCA [65]. tDNA was transcribed in vitro with T7-RNA polymerase (Fermentas) in the presence of 5-mM GMP (Sigma-Aldrich) and purified by using denaturing polyacrylamide gel electrophoresis (PAGE). Thereafter, tRNAs were denatured at 95°C for 2 min and refolded by cooling to 22°C for 3 min and incubated for 5 min at 37°C prior to transfection. tRNAs were stored at –80°C until further use [65]. The full-length tRNA^Thr^(CGU) sequence was as follows (5′ to 3′): GGCGCGGTGGCCAAGTGGTAAGGCGTCGGTCTCGTAAACCGAAGATCRCGGGTTCGAACCCCGTCCGTGCCTCCA.

tRNAs were transfected with Lipofectamine 2000 (Life Technologies) [65]. 38-ng or 150-ng tRNAs together with 600-ng plasmid DNA were incubated with 5 μl Lipofectamine 2000 in 100 μl Opti-MEM (Gibco) at 22°C for 30 min and added to subconfluent cells in 3.5-cm dishes. A detailed protocol is available at protocols.io (https://doi.org/10.17504/protocols.io.hetb3en).

CF patient–derived primary HBE cells (patient 1, ΔF508/ΔF508; patient 2, ΔF508/G551D; patients 3 and 4, ΔF508/3849 + 10kbC>T) and pulmonary tissue from a non-CF individual were kindly provided by Raymond Frizzell and Matthew Glover (University of Pittsburgh, Pittsburgh, US). Cells were isolated after informed patient consent (HSTB, University of Pittsburgh, IRB approval #0506140) and lung transplantation at the Human Airway Cell Core of the University of Pittsburgh, cultivated on transwell filters at 37°C and 5% CO_2_, trypsinized, pelleted, and stored in RNAlater (Ambion) at –80°C until further use. Total RNA from various human tissues was purchased from commercially available sources: brain (no. 540157), heart (no. 540165), and kidney (no. 540169) from Agilent and colon (no. 636553), pancreas (no. 636577), and salivary gland (no. 636552) from TAKARA/ClonTech.

### Antibodies

The following antibodies were used in this study: mouse anti-CFTR NBD1 (660; dilution 1:1,000), mouse anti-CFTR NBD2 (596; dilutions 1:100 and 1:2,500), and mouse anti-CFTR NBD2 (769; dilution 1:100) (all kindly provided by John R. Riordan and Tim Jensen, University of North Carolina, Chapel Hill, US, and Cystic Fibrosis Foundation Therapeutics, Bethesda, US), anti-CFTR NBD1 (Mr. Pink), rabbit anti-neomycin phosphotransferase II (anti-NPT; dilution 1:2,000; Merck Millipore, no. H06-747), mouse anti–β-actin (anti-ACTB; dilution 1:4,000; Sigma-Aldrich, no. A228), anti–β-catenin (dilution 1:100; Zymed), goat anti–mouse-HRP (dilution 1:10,000; BioRad, no. 170–5047), mouse anti-HA (dilution 1:1,500; Covance, no. MMS-101P), goat anti–rabbit-HRP (dilution 1:3,000; BioRad, no. 170–5046), and goat anti-mouse and goat anti-rabbit Alexa fluor-conjugated secondary antibodies (1:1,000; Molecular Probes, Inc.).

### Patch-clamp experiments

CFTR Cl^-^ channels were recorded in excised inside-out membrane patches by using an Axopatch 200B patch-clamp amplifier and pCLAMP software (both from Molecular Devices) as described previously [64, 66]. The pipette (extracellular) solution contained: 140 mM N-methyl-D-glucamine (NMDG), 140 mM aspartic acid, 5 mM CaCl_2_, 2 mM MgSO_4_, and 10 mM N-tris[hydroxymethyl]methyl-2-aminoethanesulfonic acid (TES), adjusted to pH 7.3 with Tris ([Cl^-^], 10 mM). The bath (intracellular) solution contained: 140 mM NMDG, 3 mM MgCl_2_, 1 mM CsEGTA, and 10 mM TES, adjusted to pH 7.3 with HCl ([Cl^-^], 147 mM; free [Ca^2+^], < 10^−8^ M) and was maintained at 37°C. A large Cl^-^ concentration gradient was imposed across the membrane patch (internal [Cl^-^] = 147 mM; external [Cl^-^] = 10 mM) and voltage was clamped at –50 mV to enhance the amplitude of CFTR channel openings.

CFTR Cl^-^ channels were activated promptly following membrane patch excision by the addition of the catalytic subunit of protein kinase A (PKA [purified from bovine heart], 75 nM; Calbiochem) and ATP (1 mM; Sigma-Aldrich) to the intracellular solution. To prevent channel rundown, PKA and ATP were added to all intracellular solutions. In this study, membrane patches contained ≤5 active channels, determined by using the maximum number of simultaneous channel openings as described previously [66].

We recorded, filtered, and digitized data as described previously [64], but additionally digitally filtered small-conductance T2562G-CFTR openings at 50 Hz prior to analysis. To measure single-channel current amplitude (*i*), Gaussian distributions were fit to current amplitude histograms. For open probability (*P*_o_) and burst analyses, lists of open and closed times were created by using a half-amplitude crossing criterion for event detection. For wild-type CFTR, T2562C, and wtl openings of T2562G-CFTR, transitions <1 ms in duration were excluded from the analysis, whereas for sc openings of T2562G-CFTR, transitions <4 ms in duration were excluded. Dwell time histograms were plotted with logarithmic *x* axes with 10 bins decade^-1^, and the maximum likelihood method was used to fit 1- or 2-component exponential functions to the data. Burst analysis was performed as described by Cai et al. [66] by using a *t*_c_ (the time that separates interburst closures from intraburst closures), which was determined from analyses of closed time histograms. The mean interburst interval (*T*_IBI_) was calculated by using the following equation [66]:

$$P_{o}=\frac{T_{b}}{\left({T_{MBD}+T}_{IBI}\right)}$$ (1)

where *T*_b_ = (mean burst duration) x (open probability within a burst). Mean burst duration (*T*_MBD_) and open probability within a burst (*P*_o(burst)_) were determined directly from experimental data by using pCLAMP software. Burst analysis was not performed on small-conductance T2562G-CFTR openings because of their small size. Only membrane patches that contained a single active CFTR Cl^-^ channel were used for burst analyses.

To investigate whether wtl and sc T2562G-CFTR channels are distinct channels that do not interact with each other, we performed a binomial analysis of single-channel data. Assuming that wtl and sc channels behave independently, in a membrane patch with *N* channels (each with an open probability of *p*), the probability of *k* channels being simultaneously open (*P*_(*k*)_) follows the binomial distribution:

$$P_{\left(k\right)}=\frac{N!}{k!(N-k)!}p^{k}\;{\left(1-p\right)}^{N-k}$$ (2)

Because of the small single-channel conductance of sc channels, we selected for analysis excised inside-out membrane patches with 1 active wtl channel and 1 active sc channel.

To perform binomial analysis on wtl channels and sc channels that exhibit different *P*_o_, we adopted the approach of Manivannan et al. [67] for cooperativity in 2-channel current amplitude histograms:

$$P_{\left(0\right)}=(1-p_{1})(1-p_{2})$$ (3)

$$P_{\left(1\right)}=p_{1}(1-p_{2})+p_{2}(1-p_{1})$$ (4)

$$P_{\left(2\right)}=p_{1}p_{2}$$ (5)

where *P*_(*0*)_, *P*_(*1*)_ and *P*_(*2*)_ are the probability of channels residing in the closed state (L_0_), open level 1 (L_1_), and open level 2 (L_2_), respectively (S6 Fig). *p*_1_ is the open probability of sc channels and *p*_2_ is the open probability of wtl channels. In practice, values of *p*_1_ and *p*_2_ were acquired from excised inside-out membrane patches containing only a single active channel by measuring open and closed times as described above. Using these values of *p*_1_ and *p*_2_, predicted values of *P*_(0)_, *P*_(1)_, and *P*_(2)_ were determined by using Eqs 3–5. These predicted values were then compared with experimental values of *P*_(*0*)_, *P*_(*1*)_, and *P*_(*2*)_, determined from the fit of Gaussian functions to single-channel current amplitude histograms (S6D Fig). If the predicted and experimental values of *P*_(*0*)_, *P*_(*1*)_, and *P*_(*2*)_ diverge from each other, the sc and wtl channels exhibit dependency (i.e., their gating behaviour demonstrates cooperativity) or these channels are nonidentical (i.e., they have different *P*_o_ values).

To distinguish between dependent and nonidentical channels, we calculated the cooperativity ratio (CR) [38]:

$$CR=\frac{{(P}_{\left(1\right)}P_{\left(1\right)})}{{(P}_{\left(0\right)}P_{\left(2\right)})}/\frac{\left(2N\right)}{\left(N-1\right)}$$ (6)

where *N* is the maximum number of active channels in the excised membrane patch (i.e., *N* = 2 for membrane patches with 1 wtl channel and 1 sc channel). When *N* = 2, if CR = 1, the channels are independent and identical; if CR > 1, the channels are nonidentical (i.e., the channels have unequal *P*_o_) and if CR < 1, the channels exhibit cooperativity [38].

### Immunocytochemistry

Well-differentiated primary cultures of CF bronchial epithelia (genotype: ΔF508/ΔF508) cultured at an air–liquid interface were infected with recombinant adenovirus serotype 5 at a multiplicity of infection of 100 for 1 h. The vectors encoded the cDNAs of CFTR constructs driven by a cytomegalovirus promotor. Four days after gene transfer, epithelia were examined by immunocytochemistry. Epithelia were fixed with 4% paraformaldehyde (Electron Microscopy Sciences), permeabilized with 0.3% Triton X-100 (Thermo Fisher Scientific) and blocked with 10% normal goat serum (Jackson Immunologicals) in SuperBlock (Thermo Fisher Scientific). The epithelia were incubated with the anti-CFTR antibodies 769 and 596 (1:100) and anti–β-catenin primary antibody (1:100), followed by Alexa Fluor–conjugated secondary antibodies (Molecular Probes).

### Western blotting

Cells were lysed for 15 min on ice in MNT buffer (20 mM MES, 100 mM NaCl, 30 mM Tris-HCl, pH 7.5, supplemented with 1% Triton X-100 and 1x complete protease inhibitor [Roche]) and centrifuged for 5 min at 14,000 x g (4°C) to remove cell debris. Equal amounts of lysates were mixed with SDS-loading buffer, incubated for 10 min at 37°C, separated by SDS-PAGE, and blotted onto a PVDF membrane (Millipore). Western blots were probed with the corresponding primary antibodies at 4°C overnight, followed by detection with HRP-labeled secondary antibodies and visualized by using ECL and the FujiFilm Las-4000 system (GE Healthcare). CFTR protein band intensities were normalized to the expression level of (i) NPT, which is also expressed as a selection marker on the same pcDNA3 plasmid, and (ii) ACTB, to account for differences in transfection efficiency and sample loading on the gel, respectively. The linear range for CFTR and NPT western blot assays ranged from 6.25 μg to 100 μg total protein lysate with *R*^*2*^ of 0.9418 and 0.9475, respectively. Data analysis was performed by using Prism 5 (GraphPad Software Inc.) software.

### Plasma membrane stability measurements

The plasma membrane stability of T2562G-CFTR was compared to that of wild-type CFTR by using a procedure conceptually similar to the protocol described in [30]. CFBE41o^-^ cells transfected with either wild-type or T2562G-CFTR were seeded into 6-well dishes (600,000 cells per well for each time point). At 70% confluency, 100 μg/ml cycloheximide (CHX) was added to all wells to inhibit de novo CFTR synthesis and cells were further incubated at 37°C to evaluate the plasma membrane stability of CFTR. At each time point, a well of cells was labeled at 4°C for 1 h with noncleavable biotin reagent (EZ-Link-Sulpho-NHS-S-S-biotin, Thermo Fisher Scientific), which does not penetrate the cell and attaches to amino groups located at the membrane surface. Cells were lysed in MNT buffer, and the biotin-labeled entities were immunoprecipitated with Streptavidin-sepharose bead conjugate (Cell Signaling) and washed twice with 10 mM Tris-HCl pH 8.5, containing 300 mM NaCl, 0.05% Triton X-100, and 0.1% SDS. The beads were boiled in SDS-loading buffer, and spotted onto PDFV membrane (Millipore) through a slot–blot manifold. CFTR-positive biotinylated conjugates were detected by using mouse anti-CFTR NBD1 antibodies. As a control, an aliquot of the beads was immunostained with β-actin antibodies; it was empty, showing the complete lysis of the cells and no retention of whole cells. The change in plasma membrane stability was determined by the time-dependent decrease of membrane localized CFTR-positive signal in the time course of incubation at 37°C and was compared to the zero time point at which de novo CFTR synthesis was inhibited.

### Thermal aggregation propensity assay

Thermoaggregation assays were performed as described previously [32]. Briefly, cells transiently transfected with different CFTR variants were lysed in MNT buffer (supplemented with 1% Triton X-100 and 1x complete protease inhibitor) 24 h after transfection. Equal amounts of cleared lysates were mixed with SDS-loading buffer and incubated for 10 min at different temperatures ranging from 37°C to 100°C. Aggregates were removed by centrifugation at 17,000 x g (4°C) for 5 min, and the remaining CFTR protein was analyzed by immunoblotting with anti-CFTR NBD2 antibody (596). The intensities of the B- and C-bands were quantified from immunoblots and fitted with the Boltzmann Sigmoidal equation by using Prism 5 software. Thermal aggregation temperature (*T*_*a*_) was defined as the temperature at which 50% of the protein remained soluble [32].

### RNA extraction, quantitative RT-PCR, and northern blotting

Total RNA was extracted by using TRI Reagent (Sigma-Aldrich) according to the manufacturer's protocol. RNA concentration was measured by using the NanoDrop photometer (PEQLAB Biotechnology), and RNA integrity was checked with the 2100 Bioanalyzer and RNA6000Nano Chips (both Agilent) or by denaturing agarose gel electrophoresis. RNA was stored at –80°C for further use.

Steady-state mRNA levels were determined by qRT-PCR. Total RNA was pretreated with DNase I (Fermentas) and reverse transcribed by using oligo-(dT)_18_ primers and Revert Aid H Minus M-MuLV Reverse Transcriptase (Fermentas). Amplification was performed in clear 96-well plates (Sarstedt) sealed with adhesive tape (Sarstedt) in a Mx3005P qPCR cycler (Agilent) by using QuantiFast SYBR Green PCR master mix (Qiagen) containing 6-carboxyl-X-rhodamine (ROX) as reference dye. CFTR and NPT were amplified with the following primer pairs (5′ to 3′): CFTR forward, CCTATGTCAACCCTCAACACG and CFTR reverse, ACTATCACTGGCACTGTTGC; NPT forward, TGCTCCTGCCGAGAAAGTAT and NPT reverse, GCTCTTCGTCCAGATCATCC. Primers were used at a final concentration of 300 nM. Relative expression levels were calculated by using the ΔΔ*C*_*T*_*-*method and normalized to NPT signals. Each reaction was performed in duplicate with the following controls included in each run: a no-template control (NTC) and a not-reverse-transcribed sample. qRT-PCR assays for CFTR and NPT displayed a linear range over 6 (*R*^*2*^, 0.9952) and 5 (*R*^*2*^, 0.9944) orders of magnitude, respectively. qPCR efficiencies were 99% (slope, 3.345) and 108% (slope, 3.145) for CFTR and NPT, respectively. Data analysis was performed by using MxPro QPCR (Agilent) and Prism 5 software.

tRNA^Thr^(CGU) levels were analyzed by using the QuantiFast SYBR Green RT-PCR kit (Qiagen) containing ROX reference dye. tRNA^Thr^(CGU) and 5S rRNA were amplified by using the following primer pair (5′ to 3′) at a final concentration of 1 μM: tRNA^Thr^(CGU) forward, GGCCAAGTGGTAAGGC and tRNA^Thr^(CGU) reverse, AGGCACGGACGGG; 5S rRNA forward, CCATACCACCCTGAACGC and 5S rRNA reverse, GTATTCCCAGGCGGTCTC. tRNA signals were normalized to 5S rRNA values.

For northern blotting, equal amounts of total RNA were separated by denaturing PAGE, in some cases stained with SYBR gold (Sigma-Aldrich) for visualization and subsequently transferred onto HyBond-N+ membrane (GE Healthcare). tRNA^Thr^(CGU) was detected by overnight hybridization at 60°C with Cy3-labeled probes complementary to the tRNA anticodon loop (5′-Cy3-CTTCGGTTTACGAGACCGACGCCTTA-3′). Blots were subsequently washed at 35°C 3 times with 6x SSC (supplemented with 0.1% SDS), followed by 1 wash with 6x SSC, 1 wash with 2x SSC, and a final wash with 0.2x SSC and imaged on the FujiFilm Las-4000 system (GE Healthcare). Intensities were normalized to 5S rRNA probed also with 5′-Cy3-labeled oligonucleotide (5′-AAGTACTAACCGCGCCCGAC-3′).

### tRNA microarrays

tRNA microarrays [68] were performed with tRNA probes covering the full-length sequence of 41 cytoplasmic tRNA species complementary to 49 nuclear-encoding tRNA families with sequences described previously [20]. Each microarray consisted of 12 identical blocks, each containing 2 probes for each tRNA (i.e., in total 24 measurements for each tRNA). Total RNA was extracted from the cells by using TRI Reagent and deacetylated for 45 min at 37°C with 100 mM Tris-HCl (pH 9.0). Fluorescent stem-loop RNA/DNA oligonucleotide [20] bearing a Cy3 or Atto647 fluorescent dye (Microsynth) was ligated overnight at 16°C with T4 DNA ligase (NEB) to all deacetylated tRNAs [68]. Ligation efficiency was analyzed by resolving the samples with denaturing 10% PAGE and detected by fluorescence (Fujifilm LAS-4000) and SYBR gold (Invitrogen) staining. Fluorescently labeled tRNAs were hybridized on the microarrays for 16 h at 60°C in the Hyb4 microarray hybridization system (Digilab). Subsequently, the microarrays were washed once in 2x SSC/0.1% SDS (50°C), once in 1x SSC/0.1% SDS (42°C) and then 3 times in 0.1x SSC (42°C) before scanning by using a GenPIX 4200A (Molecular Devices) scanner [68]. Analysis of the scanned arrays was performed by using the GenPix Pro 7 (Molecular Devices) software. For normalization, identical amounts of in vitro–synthesized tRNA standards (i.e., *Escherichia coli* tRNA^Lys^(UUU), *E*. *coli* tRNA^Tyr^(AUA), and *Saccharomyces cerevisiae* tRNA^Phe^(GAA)) were added to each total RNA sample prior to deacetylation. Quantification was performed by normalizing the median of the Cy3-tRNA signal of each tRNA species to the corresponding Atto647-labeled HEK tRNA signal. Note that the human genome lacks a gene for tRNA^Thr^(GGU) reading the Thr codon ACC (http://gtrnadb.ucsc.edu), which is likely to be decoded by tRNA^Thr^(IGU) via deamination of adenine (A) to inosine (I) in the tRNA wobble position [69].

Absolute tRNA levels of HeLa cells were determined by using the tRNA microarrays. Gel-purified tRNA from the total RNA was ligated to increasing concentrations of the Cy3-labeled fluorescent stem-loop RNA/DNA oligonucleotide. Only values in the linear range (0.57 μM, 1.13 μM, and 2.25 μM) were considered from several microarrays. A detailed protocol is available at protocols.io (https://doi.org/10.17504/protocols.io.hfcb3iw).

### tRNA silencing with shRNA

tRNA silencing was performed by using shRNAs targeting the anticodon loop of tRNA^Thr^(CGU) [70]. In brief, shRNAs were cloned into the pSUPER vector (Oligoengine) by using BglII and HindIII restriction sites according to the manufacturer's instructions. shRNA bearing pSUPER plasmids (1 μg) were cotransfected together with CFTR constructs (1 μg) into subconfluent HeLa cells (3.5-cm dish) by using PEI. Mock-transfected cells were cotransfected with CFTR constructs and an empty pSUPER vector. Two different shRNA sequences targeting tRNA^Thr^(CGU) were used (5′ to 3′):

shThr1, GGCGTCGGTCTCGTAAACCGAAGTTCAAGAGACTTCGGTTTACGAGACCGACGCC and shThr2, GTGGTAAGGCGTCGGTCTCGTAATTCAAGAGATTACGAGACCGACGCCTTACCAC (underlined nucleotides denote the sense tRNA^Thr^(CGU) target sequence). Cells were analyzed for CFTR protein level or tRNA^Thr^(CGU) level 48 h after transfection. A detailed protocol is available at protocols.io (https://doi.org/10.17504/protocols.io.hgfb3tn).

### Ribosome profiling and read calibration

Approximately 5 million CFBE41o^-^ cells, in 3 independent biological replicates, were used to isolate mRNA-bound ribosome complexes, followed by extraction of RNase I digestion-derived, ribosome-protected fragments (RPFs) as described in [14]. Cells were collected by flash-freezing without preincubation with antibiotics. CHX (100 μg/ml) and harringtonine (2 μg/ml) were present in the lysis buffer and the sucrose gradient buffer to prevent ribosome dissociation in the postprocessing steps. The cDNA libraries from RPFs were prepared by using a modified protocol for miRNA with direct ligation of the adapters and were sequenced with TruSeq SBS kits (Illumina) on a HiSeq2000 (Illumina) machine. Sequenced reads were trimmed by using *fastx-toolkit* (0.0.13.2; quality threshold: 20), and sequencing adapters were cut by using *cutadapt* (1.2.1; minimal overlap: 1 nt). Processed reads were uniquely mapped to the human genome (GRCh37) by using Bowtie (0.12.9), allowing a maximum of 2 mismatches (parameter settings: -l 16 -n 1 -e 50 -m 1—strata—best y). RPFs were binned in groups of equal read length, and each group was aligned to the start or stop codons as described in [21, 71]. Taking into account that the P site of the ribosome covers the start codon, for each read length we calculated the distance between the middle nucleotide in the A site and 5′ of the read by using this distance to determine the center of each A site codon along each mRNA. We used 5′ calibration of the reads, as the RNase I cleavage was more variable on the 3′ side of the ribosome-protected fragment, which is consistent with prior studies [21, 71]. The majority of our sequence reads were 27–29 nts as expected. The ribosome dwelling occupancy per codon was calculated as described [40]. The reads over each position *i* in a gene were normalized to the average number of footprints across this gene. These ratios were then averaged across all genes to give the ribosome dwelling occupancy of a given codon in the transcriptome. The Met codon was excluded from these calculations because N-terminal Met is influenced by the presence of harringtonine used in the processing buffers. The first 51 nt were excluded from the calculations to avoid depletion of ribosomes at the beginning of genes by runoff elongation during cell harvesting [40].

### Statistical analysis and data deposition

If not stated otherwise, results are expressed as means ± SEM of *n* observations. Sample sizes of cell analyses were selected to demonstrate reproducibility among independent biological replicates and with adequate power to resolve significant differences among conditions. Patient samples were blindly allocated during experiments without prior knowledge of genotype; each separate patient-derived primary HBE cell sample was considered a single biological replicate. Differences between groups were evaluated by using 2-tailed Student *t* test implemented in Prism 5 or SigmaPlot 12 (Systat Software Inc.) software. Differences were considered statistically significant when *P* < 0.05.

tRNA microarray data have been deposited with the Gene Expression Omnibus (GEO) under the accession number GSE53991. The sequencing data were also submitted to GEO under the accession number GSE74365.

## Supporting information

S1 Fig Localization of T2562G sSNP in CFTR structural model and its effect on CFTR expression and alternative mRNA splicing. (**A**) Location of the sSNPs studied on a CFTR structural model [72]. Abbreviations: nucleotide-binding domains 1 and 2, NBD1 (light blue) and NBD2 (pink), membrane-spanning domains 1 and 2, MSD1 (wheat) and MSD2 (light green), and regulatory R-domain (grey). Note that T2562G is located in the R-domain. (**B**) Representative immunoblots of CFTR variants transiently expressed for 24 h and used for the quantification shown in Fig 1B. CFTR was probed with the anti-CFTR NBD2 (596) antibody. Total CFTR expression (sum of bands B and C) was normalized to the expression of neomycin phosphotransferase (NPT), encoded on the same plasmid; NPT served as an internal transfection control and β-actin (ACTB) as a loading control. Numbers on the left indicate molecular mass standards, B and C denote immature and mature CFTR protein, respectively and mock denotes transfection with empty plasmid expressing NPT only. (**C, D**) Representative analysis of the effects of the T2562G sSNP on exon 15 splicing using Bioanalyzer and DNA1000 Chip (Agilent), presented as capillary electrophoregram (C) and band intensity profile (D). The chip runs with 15 bp and 1500 bp standards marked as lower (LM) and upper marker (UM), respectively. pET01 denotes vector with the splicing minigene cassette [27, 73] without exon 15. Correctly spliced mRNA with full-length exon 15 and the alternatively spliced product (skipped) are designated. (**E**) Quantification of the full-length and skipped exon from the electrophoregrams in C and D. Data are means ± SEM (*n* = 3). The underlying data of panel E can be found in S1 Data.
(TIF)

S2 Fig T2562G sSNP increases the susceptibility of CFTR to proteolysis, ubiquitination and CHIP binding. (**A**) Ubiquitination of T2562G-CFTR compared to wild-type CFTR. Immunoprecipitated CFTR was probed with anti-CFTR antibodies, while CFTR-ubiquitin-HA conjugates were detected with an anti-HA antibody. Input denotes the cell lysates prior to immunoprecipitation probed with an anti-CFTR antibody. Representative blots of three biological replicates are shown. (**B**) Quantification of the ubiquitinated CFTR species. The ubiquitination of wild-type was set as 100%. Data are means ± SEM (n = 4); *, *P* < 0.05 versus wild-type CFTR. (**C**) Proteasome inhibition results in increased protein expression of T2562G-CFTR. HeLa cells expressing wild-type and T2562G-CFTR were incubated with and without the proteasomal inhibitor MG132 and analyzed by immunoblotting with the anti-CFTR NBD2 (596) antibody. (**D**) Quantification of CFTR protein expression in MG132-treated cells from C normalized to NPT expression to account for differences in transfection. The expression of each variant without MG132 was set as 100%. Data are means ± SEM (*n* = 3–4); * *P* < 0.05 versus wild-type CFTR. (**E**) Co-immunoprecipitation of CHIP and RMA1 with CFTR variants expressed in HeLa cells using an anti-CFTR antibody and probed with anti-CHIP and anti-RMA1 antibodies. Input denotes the cell lysates prior to co-immunoprecipitation probed with anti-CFTR, anti-ACTB, anti-CHIP or anti-RMA1 antibodies. Note that to avoid overloading the gel only 5% of the input amount was loaded for visualization. Equal amounts of cell lysates were used for each CFTR variant as demonstrated by the ACTB immunostaining. (**F, G**) Quantification of CHIP and RMA1 bound to CFTR variants from the immunoblots in E. The amount of co-immunoprecipitated CHIP and RMA1 were normalized to the amount of immunoprecipitated CFTR variant (i.e. the sum of bands B and C), which was arbitrarily set to 1. Data are means ± SEM (*n* = 4–5); * *P* < 0.05 versus wild-type CFTR. (**H**) Representative autoradiogram of pulse-chase analysis of CFTR variants. (**I**) Quantification of the pulse-chase autoradiograms represented as average disappearance of band B (maturation and/or degradation) and formation of band C. Results are plotted as percent of band B density at the 0 min time point and are means ± SEM (*n* = 5–7); *P* = 0.7123 for time point 240 min. (**J**) Quantification of the amount of membrane-localized CFTR upon inhibition of de novo synthesis at 37°C using surface biotinylation of non-permeabilized cells. Data are means ± SEM (*n* = 3); * *P* < 0.05 versus wild-type CFTR. The underlying data of panels B, D, F, G and I can be found in S1 Data.
(TIF)

S3 Fig The T2562G sSNP increases the proteolytic stability of CFTR. (**A**) Representative immunoblots of limited trypsin digestion of wild-type and T2562G-CFTR in semi-intact HeLa cells probed with anti-CFTR NBD2 (596) antibody. The positions of the band B and C forms of CFTR protein are indicated; NBD2 denotes characteristic nucleotide-binding domain 2 (NBD2)-containing fragments. (**B**) Quantification of full-length CFTR (sum of bands B and C) from panel A relative to untreated samples (set to 100%). Data are means ± SD (*n* = 2). The underlying data of panel B can be found in S1 Data.
(TIF)

S4 Fig Small-conductance (sc) and wild-type-like (wtl) openings of T2562G-CFTR occur either together or in isolation. (**A-C**) Representative single-channel recordings of T2562G-CFTR in excised inside-out membrane patches from HeLa cells transiently expressing T2562G-CFTR. In A, the recordings were made in the absence and presence of ATP (1 mM) and PKA (75 nM) in the intracellular solution. Neither sc nor wtl channel openings of T2562G-CFTR were observed in the absence of ATP and PKA (basal). However, two T2562G-CFTR channels, one sc and one wtl, were activated following phosphorylation with PKA. In panels B and C, the recordings were made in the presence of ATP (1 mM) and PKA (75 nM) in the intracellular solution. In B, the membrane patch contained only one active wtl channel, whereas in panel C the membrane patch contained only one active sc channel. Dashed lines indicate the closed channel state and downward deflections correspond to channel openings. For presentation purposes, all single-channel recordings were digitally filtered at 50 Hz.
(TIF)

S5 Fig T2562G-CFTR exhibits two populations of channel openings distinguished by small- and wild-type-like-conductances. (**A**) Representative single-channel current amplitude histograms of wild-type, T2562G- and T2562C-CFTR. The histograms were made from 10-s long recordings filtered at 50 Hz using the experimental conditions shown in Fig 3. The continuous lines are the fit of Gaussian distributions to the data and the vertical dashed lines indicate the positions of the sc- and wtl-conductance openings of T2562G-CFTR; the closed channel amplitude is shown on the left. (**B**) Single-channel current-voltage (i-V) relationships of wild-type, T2562G- and T2562C-CFTR. Data are means ± SEM (*n* = 6–10). Continuous lines are the fit of first order linear regression functions to mean data. Note that the i-V relationship of T2562C is obscured by that of wild-type CFTR. (**C**) Single-channel conductance (γ) of wild-type, T2562G- and T2562C-CFTR determined from the slope of the i-V relationships in B; * *P* < 0.05 versus wild-type CFTR. The underlying data of panels B and C can be found in S1 Data.
(TIF)

S6 Fig Binomial analysis demonstrates that sc and wtl channels of T2562G-CFTR are independent channel populations. (**A**) A representative 3-minute single-channel recording of T2562G-CFTR in an excised inside-out membrane patch from a CHO cell commenced following full channel activation. This membrane patch contained two channels, one with sc and the other with wtl openings. ATP (1 mM) and PKA (75 nM) were continuously present in the intracellular solution. The 2-s long segments labelled 1–4 indicated by bars are displayed on an expanded time scale beneath the 3-minute recording and the segment labelled 5 indicates the high resolution recording displayed in B. Arrows and dashed lines indicate the closed channel state and downward deflections correspond to channel openings. For presentation purposes, single-channel records were filtered digitally at 50 Hz. (**B-D**) Binomial analysis of the T2562G channel recording displayed in A. (**B**) High resolution T2562G-CFTR channel recording. Letters and arrows indicate openings to different channel levels. Abbreviations: L_0_, both channels closed; L_1-sc_, one sc channel open; L_1-wtl_, one wtl channel open; L_2-sc+wtl_, one sc channel and one wtl channel open. (**C**) The single-channel current amplitude histogram for the T2562G-CFTR recording shown in B. The continuous line is the fit of a Gaussian distribution to the data (R = 0.94) and the dashed lines show the individual components of the Gaussian function. The vertical dashed lines indicate the positions of the different channel levels with the closed channel level (L_0_) shown on the left. (**D**) Probability (*P*_(k)_) of the wtl and sc T2562G-CFTR channels residing in either the closed level (L_0_), open level 1, (L_1_) or open level 2 (L_2_). Columns represent predicted *P*_(k)_ values calculated using mean *P*_o_ values from membrane patches containing either one wtl or one sc channel (wtl, *n* = 4; sc, *n* = 3). Symbols and error bars show experimental *P*_(k)_ values calculated using *P*_o_ values from three membrane patches containing one wtl channel and one sc channel. The underlying data of panel D can be found in S1 Data.
(TIF)

S7 Fig Other synonymous mutations at position T2562 do not alter CFTR expression. (**A**) Representative immunoblot analysis of CFTR T2562 sSNP variants in HeLa cells. NPT served as a transfection control. (**B**) Quantification of steady-state protein expression levels from the immunoblots in A. CFTR expression (i.e., the sum of bands B and C) was normalized to that of NPT to correct for differences in transfection efficiencies. The level of wild-type CFTR (ACT codon) was set to 100%. Data are means ± SEM (*n* = 8); * *P* < 0.05 versus wild-type CFTR. (**C**) qRT-PCR quantification of mRNA expression levels for CFTR T2562 codon variants at 24 h after transfection. The mRNA level of wild-type CFTR (ACT codon) was set to 100%. Data are means ± SEM (*n* = 3–7). (**D, E**) Thermal aggregation propensity analysis for CFTR sSNP variants. Thermal aggregation temperatures (*T_a_*) are expressed as means ± SEM (*n* = 2–5); * *P* < 0.05 versus wild-type CFTR. (**F**) Quantification of the expression of CFTR codon variants in CHO cells as described in panel B. The level of wild-type CFTR was set to 100%. Data are means ± SEM (*n* = 4); ** *P* < 0.01. The underlying data of panels B-C and E-F can be found in S1 Data.
(TIF)

S8 Fig CFBE41o^-^ and CF patient-derived HBE cells display similar tRNA abundance. (**A**) Microarray analysis of relative tRNA abundance of all tRNAs in CFBE41o^-^ and four CF patient-derived HBE cells were measured relative to the values for HeLa tRNAs. tRNA probes are depicted with their cognate codon and the corresponding amino acid; tRNAs^Thr^ highlighted by the box are presented in Fig 4D. Data are means ± SEM (*n* = 3–5). (**B, C**) Correlation between codon usage and absolute tRNA concentration for HeLa (B) and CFBE41o^-^ cells (C). (**D**) Differences in tRNA abundance between CFBE41o^-^ and HeLa cells. Negative sign denotes that the corresponding tRNAs are more abundant in CFBE41o^-^ cells and vice versa. Inset, box-plot with designated outliers. The underlying data of panels A-D can be found in S1 Data.
(TIF)

S9 Fig Modulation of the tRNA^Thr^(CGU) pool does not alter global translation in HeLa cells. (**A**) Northern blot of HeLa cells transfected with increasing amounts (ng/well) of in vitro transcribed tRNA^Thr^(CGU). 5S rRNA served as a loading control. Lane 1 (0 ng tRNA) represents nontransfected HeLa. (**B**) Transfection of tRNA^Thr^(CGU) does not alter the cellular tRNA pool. (C) Addition of tRNAThr(CGU) (150 ng) does not change polysomal profiles. Mock denotes control HeLa cells. M:P denotes the ratio between monosomal (M) and polysomal (P) peaks. (**D**) tRNA^Thr^(CGU) is increased in both M and P fractions. The ratio of tRNA^Thr^(CGU) in M and P to which 150 ng tRNA^Thr^(CGU) were added was quantified by qRT-PCR, normalized to 5S rRNA and expressed as fold-change ± SD compared to tRNA^Thr^(CGU) levels in polysomal and monosomal fractions of untreated control HeLa (mock) cells. Values are means ± SEM (*n* = 3). (**E**) Reduction of tRNA^Thr^(CGU) levels (reduced to ~75 or ~60%) using two shRNAs (shThr1 and shThr2), measured with qRT-PCR and normalized to 5S rRNA. Values are means ± SEM (*n* = 3). (**F**) Reduction of tRNA^Thr^(CGU) decreases the protein levels of both wild-type and T2562G-CFTR. Representative immunoblot of four biological replicates. The positions of the band B and C forms of CFTR protein are indicated. NPT served as an internal transfection control and ACTB as a loading control. (**G**) Transfection with shRNAs (shThr1 and shThr2) to reduce tRNA^Thr^(CGU) does not alter polysomal profiles. Note that tRNA^Thr^(CGU) can only be partially downregulated as complete knockdown would perturb global translation and cell viability. The underlying data of panels D and E can be found in S1 Data.
(TIF)

S1 Table Synonymous mutations detected in CF patients. (DOCX)

S2 Table Frequency of observed small-conductance openings of T2562G-CFTR. (DOCX)

S3 Table Frequency of usage of different codons in CFTR compared to their usage in the human genome. (DOCX)

S1 Text Supporting methods. (DOCX)

S1 Data Supplementary data. (XLSX)

## Abbreviations

ABC: ATP-binding cassette
ACTB: β-actin
CF: cystic fibrosis
CFTR: cystic fibrosis transmembrane conductance regulator
CHIP: C-terminus of Hsc70-interacting protein
CR: cooperativity ratio
ER: endoplasmic reticulum
HBE: human bronchial epithelial
IBI: interburst interval
Met_i_: initiator tRNA^Met^
MBD: mean burst duration
MSD: membrane-spanning domain
NB: northern blot
NBD: nucleotide-binding domain
ND: not determined
NPT: neomycin phosphotransferase
PKA: protein kinase A
*P*_o_: open probability
qRT-PCR: quantitative real-time PCR
RNF5: RING Finger Protein 5
sc: small conductance
sSNP: synonymous single nucleotide polymorphism
WB: western blot or immunoblot
wtl: wild-type–like
