# Supplementary figures and images for: Alteration of protein function by a silent polymorphism linked to tRNA abundance
Source: PLoS Biol. 2017 May 16;15(5):e2000779. doi: 10.1371/journal.pbio.2000779 (PMC5433685; doi:10.1371/journal.pbio.2000779)

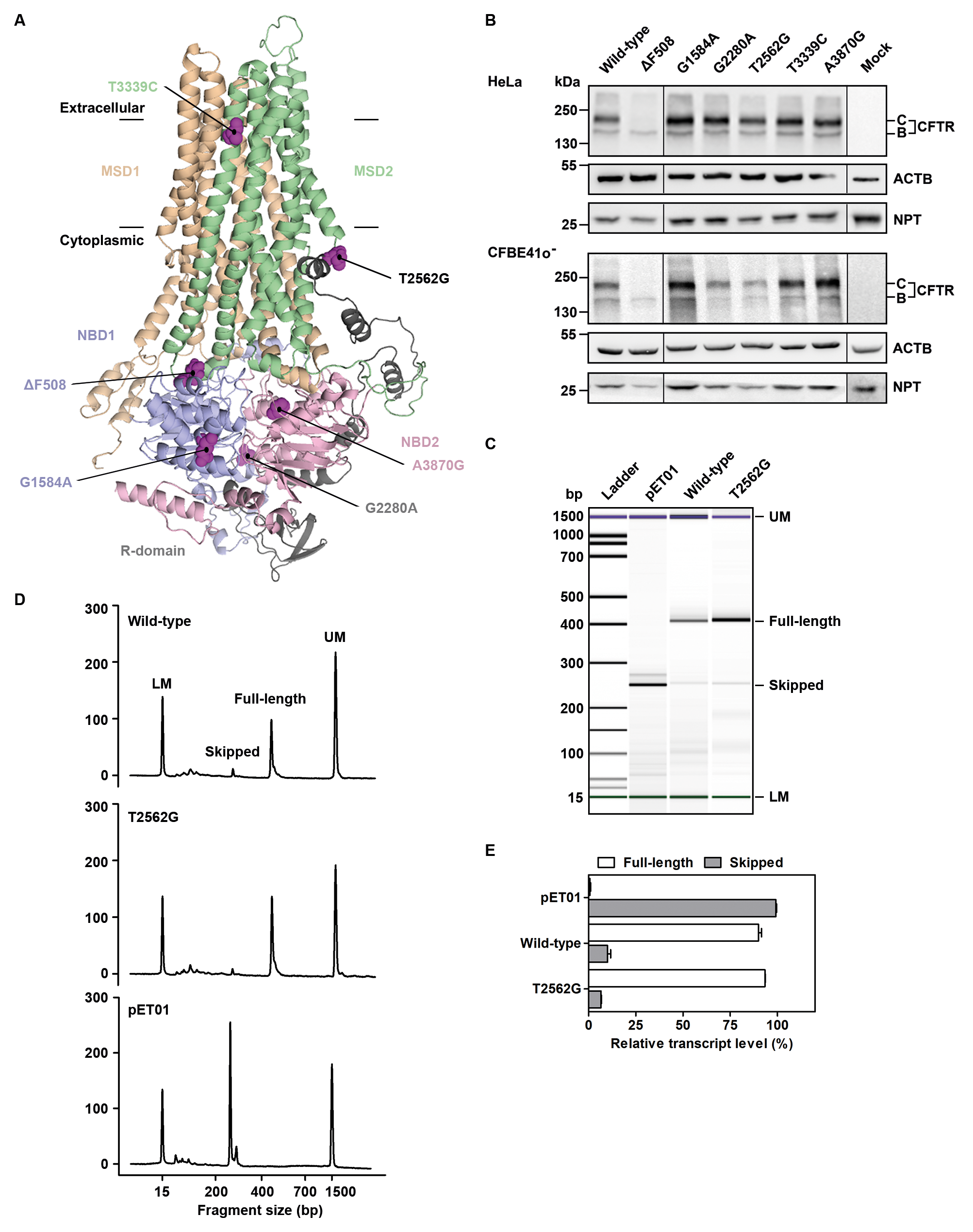

Supplement: S1 Fig — (A) Location of the sSNPs studied on a CFTR structural model [72]. Abbreviations: nucleotide-binding domains 1 and 2, NBD1 (light blue) and NBD2 (pink), membrane-spanning domains 1 and 2, MSD1 (wheat) and MSD2 (light green), and regulatory R-domain (grey). Note that T2562G is located in the R-domain. (B) Representative immunoblots of CFTR variants transiently expressed for 24 h and used for the quantification shown in Fig 1B. CFTR was probed with the anti-CFTR NBD2 (596) antibody. Total CFTR expression (sum of bands B and C) was normalized to the expression of neomycin phosphotransferase (NPT), encoded on the same plasmid; NPT served as an internal transfection control and β-actin (ACTB) as a loading control. Numbers on the left indicate molecular mass standards, B and C denote immature and mature CFTR protein, respectively and mock denotes transfection with empty plasmid expressing NPT only. (C, D) Representative analysis of the effects of the T2562G sSNP on exon 15 splicing using Bioanalyzer and DNA1000 Chip (Agilent), presented as capillary electrophoregram (C) and band intensity profile (D). The chip runs with 15 bp and 1500 bp standards marked as lower (LM) and upper marker (UM), respectively. pET01 denotes vector with the splicing minigene cassette [27, 73] without exon 15. Correctly spliced mRNA with full-length exon 15 and the alternatively spliced product (skipped) are designated. (E) Quantification of the full-length and skipped exon from the electrophoregrams in C and D. Data are means ± SEM (n = 3). The underlying data of panel E can be found in S1 Data. (TIF) [file pbio.2000779.s001.tif]

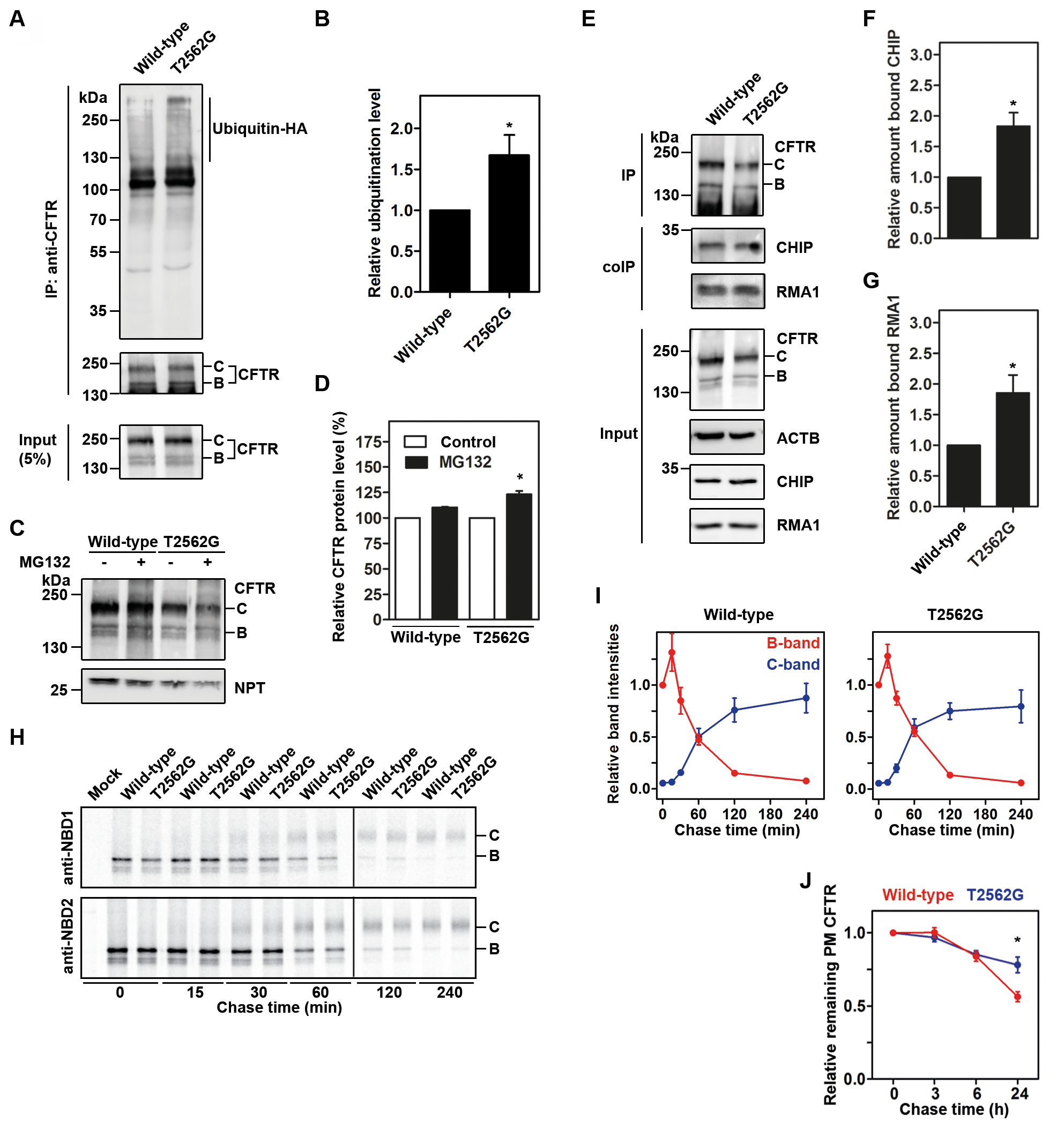

Supplement: S2 Fig — (A) Ubiquitination of T2562G-CFTR compared to wild-type CFTR. Immunoprecipitated CFTR was probed with anti-CFTR antibodies, while CFTR-ubiquitin-HA conjugates were detected with an anti-HA antibody. Input denotes the cell lysates prior to immunoprecipitation probed with an anti-CFTR antibody. Representative blots of three biological replicates are shown. (B) Quantification of the ubiquitinated CFTR species. The ubiquitination of wild-type was set as 100%. Data are means ± SEM (n = 4); *, P < 0.05 versus wild-type CFTR. (C) Proteasome inhibition results in increased protein expression of T2562G-CFTR. HeLa cells expressing wild-type and T2562G-CFTR were incubated with and without the proteasomal inhibitor MG132 and analyzed by immunoblotting with the anti-CFTR NBD2 (596) antibody. (D) Quantification of CFTR protein expression in MG132-treated cells from C normalized to NPT expression to account for differences in transfection. The expression of each variant without MG132 was set as 100%. Data are means ± SEM (n = 3–4); * P < 0.05 versus wild-type CFTR. (E) Co-immunoprecipitation of CHIP and RMA1 with CFTR variants expressed in HeLa cells using an anti-CFTR antibody and probed with anti-CHIP and anti-RMA1 antibodies. Input denotes the cell lysates prior to co-immunoprecipitation probed with anti-CFTR, anti-ACTB, anti-CHIP or anti-RMA1 antibodies. Note that to avoid overloading the gel only 5% of the input amount was loaded for visualization. Equal amounts of cell lysates were used for each CFTR variant as demonstrated by the ACTB immunostaining. (F, G) Quantification of CHIP and RMA1 bound to CFTR variants from the immunoblots in E. The amount of co-immunoprecipitated CHIP and RMA1 were normalized to the amount of immunoprecipitated CFTR variant (i.e. the sum of bands B and C), which was arbitrarily set to 1. Data are means ± SEM (n = 4–5); * P < 0.05 versus wild-type CFTR. (H) Representative autoradiogram of pulse-chase analysis of CFTR variants. (I) Quantification of [file pbio.2000779.s002.tif]

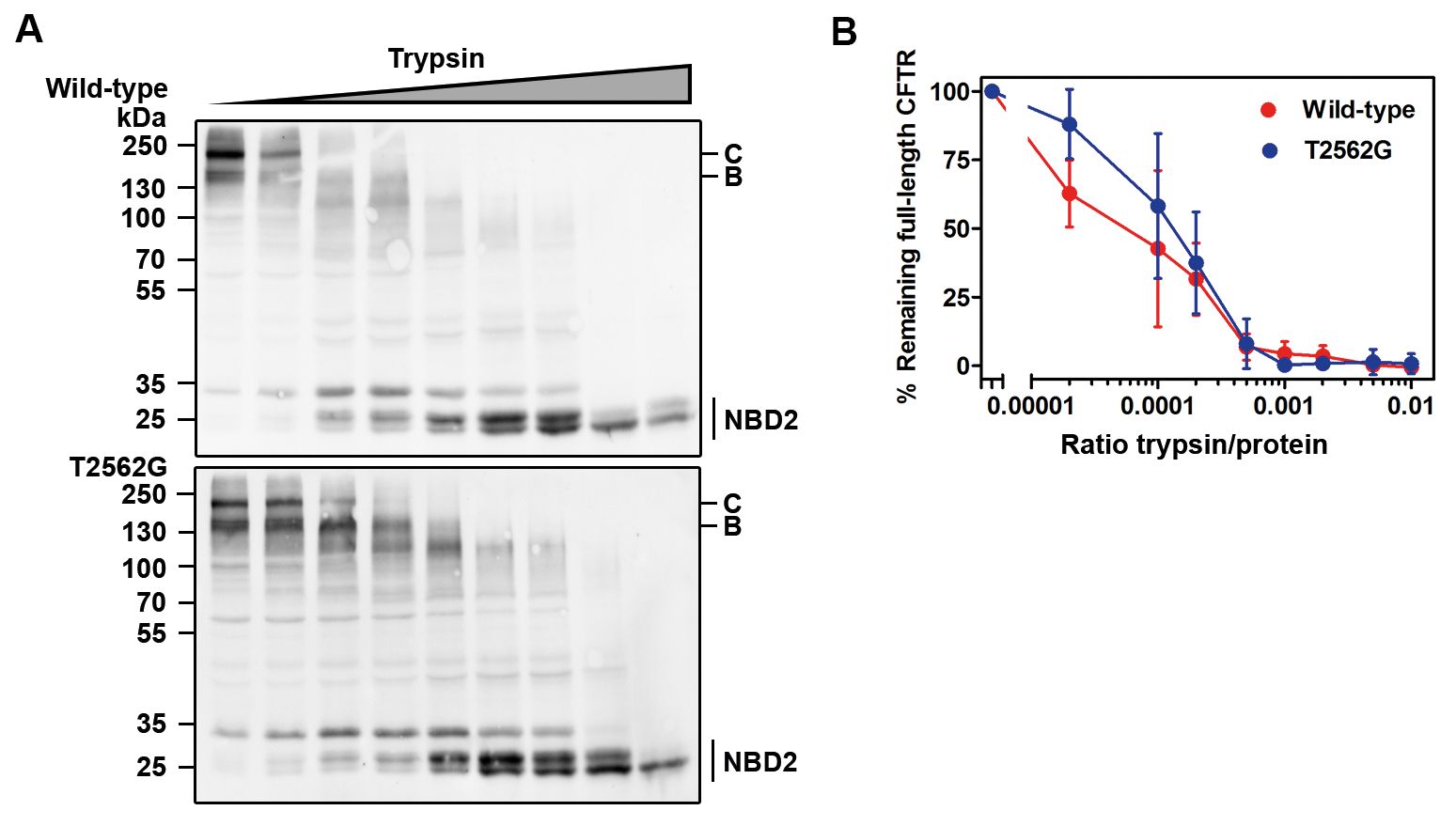

Supplement: S3 Fig — (A) Representative immunoblots of limited trypsin digestion of wild-type and T2562G-CFTR in semi-intact HeLa cells probed with anti-CFTR NBD2 (596) antibody. The positions of the band B and C forms of CFTR protein are indicated; NBD2 denotes characteristic nucleotide-binding domain 2 (NBD2)-containing fragments. (B) Quantification of full-length CFTR (sum of bands B and C) from panel A relative to untreated samples (set to 100%). Data are means ± SD (n = 2). The underlying data of panel B can be found in S1 Data. (TIF) [file pbio.2000779.s003.tif]

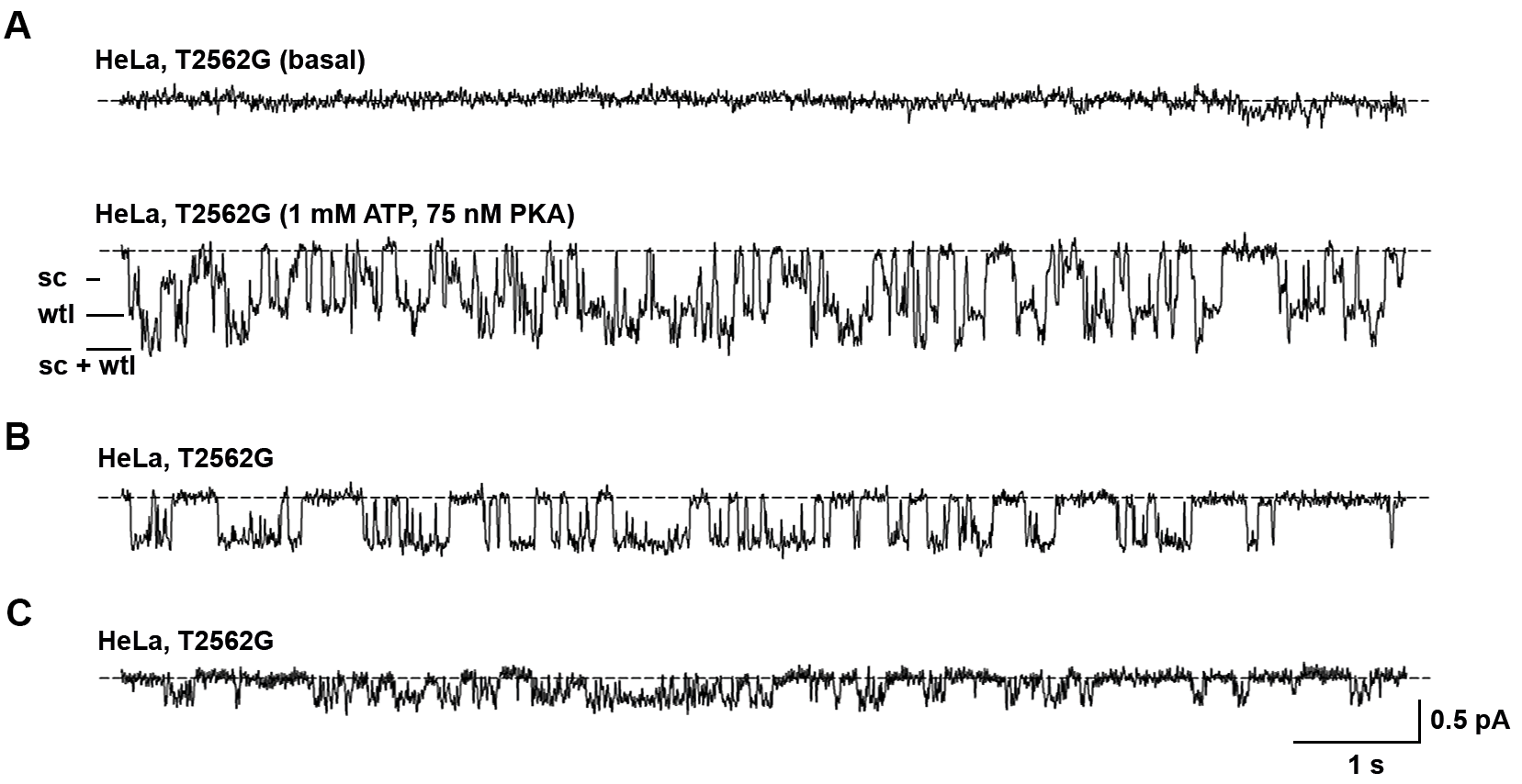

Supplement: S4 Fig — (A-C) Representative single-channel recordings of T2562G-CFTR in excised inside-out membrane patches from HeLa cells transiently expressing T2562G-CFTR. In A, the recordings were made in the absence and presence of ATP (1 mM) and PKA (75 nM) in the intracellular solution. Neither sc nor wtl channel openings of T2562G-CFTR were observed in the absence of ATP and PKA (basal). However, two T2562G-CFTR channels, one sc and one wtl, were activated following phosphorylation with PKA. In panels B and C, the recordings were made in the presence of ATP (1 mM) and PKA (75 nM) in the intracellular solution. In B, the membrane patch contained only one active wtl channel, whereas in panel C the membrane patch contained only one active sc channel. Dashed lines indicate the closed channel state and downward deflections correspond to channel openings. For presentation purposes, all single-channel recordings were digitally filtered at 50 Hz. (TIF) [file pbio.2000779.s004.tif]

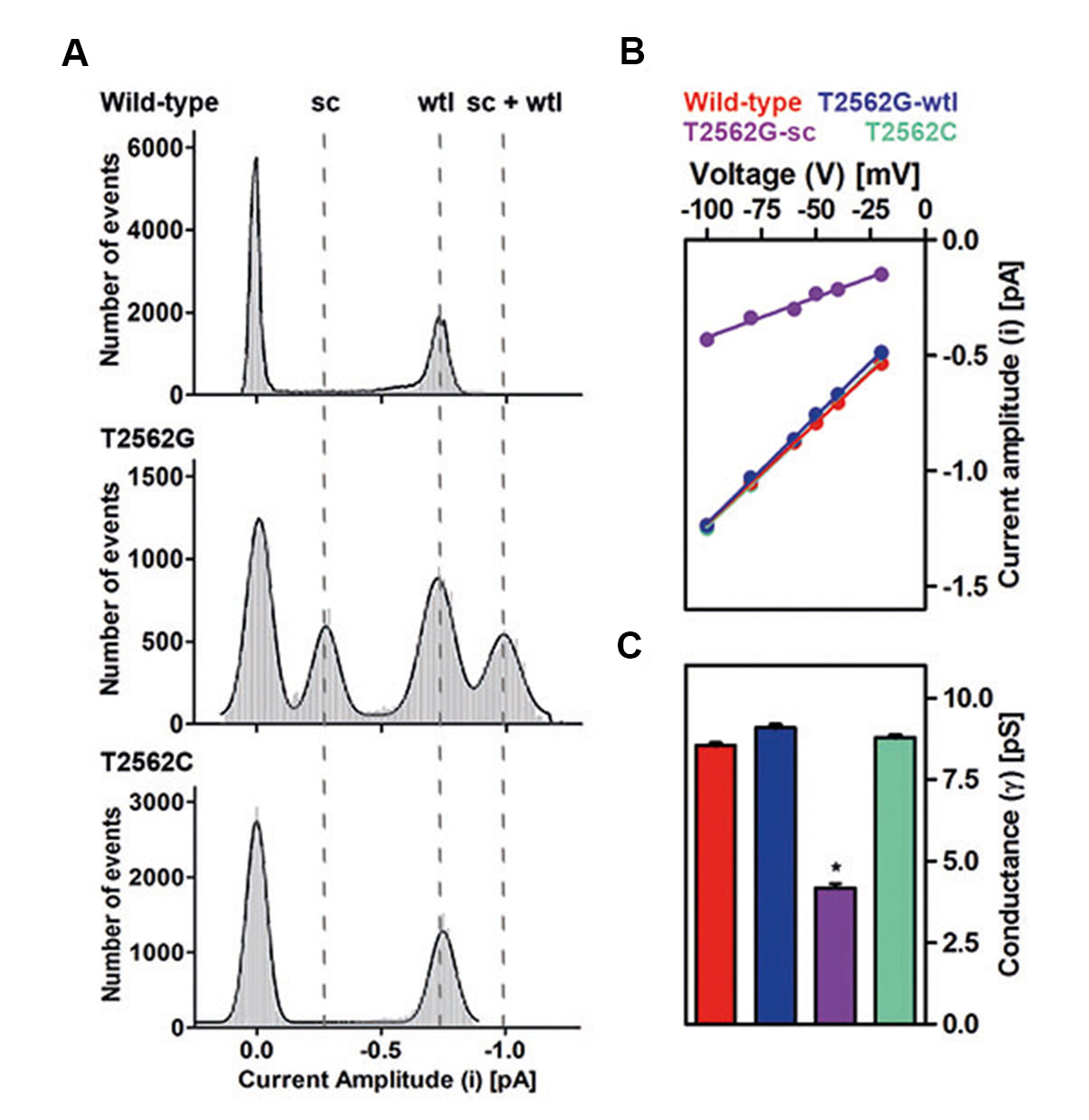

Supplement: S5 Fig — (A) Representative single-channel current amplitude histograms of wild-type, T2562G- and T2562C-CFTR. The histograms were made from 10-s long recordings filtered at 50 Hz using the experimental conditions shown in Fig 3. The continuous lines are the fit of Gaussian distributions to the data and the vertical dashed lines indicate the positions of the sc- and wtl-conductance openings of T2562G-CFTR; the closed channel amplitude is shown on the left. (B) Single-channel current-voltage (i-V) relationships of wild-type, T2562G- and T2562C-CFTR. Data are means ± SEM (n = 6–10). Continuous lines are the fit of first order linear regression functions to mean data. Note that the i-V relationship of T2562C is obscured by that of wild-type CFTR. (C) Single-channel conductance (γ) of wild-type, T2562G- and T2562C-CFTR determined from the slope of the i-V relationships in B; * P < 0.05 versus wild-type CFTR. The underlying data of panels B and C can be found in S1 Data. (TIF) [file pbio.2000779.s005.tif]

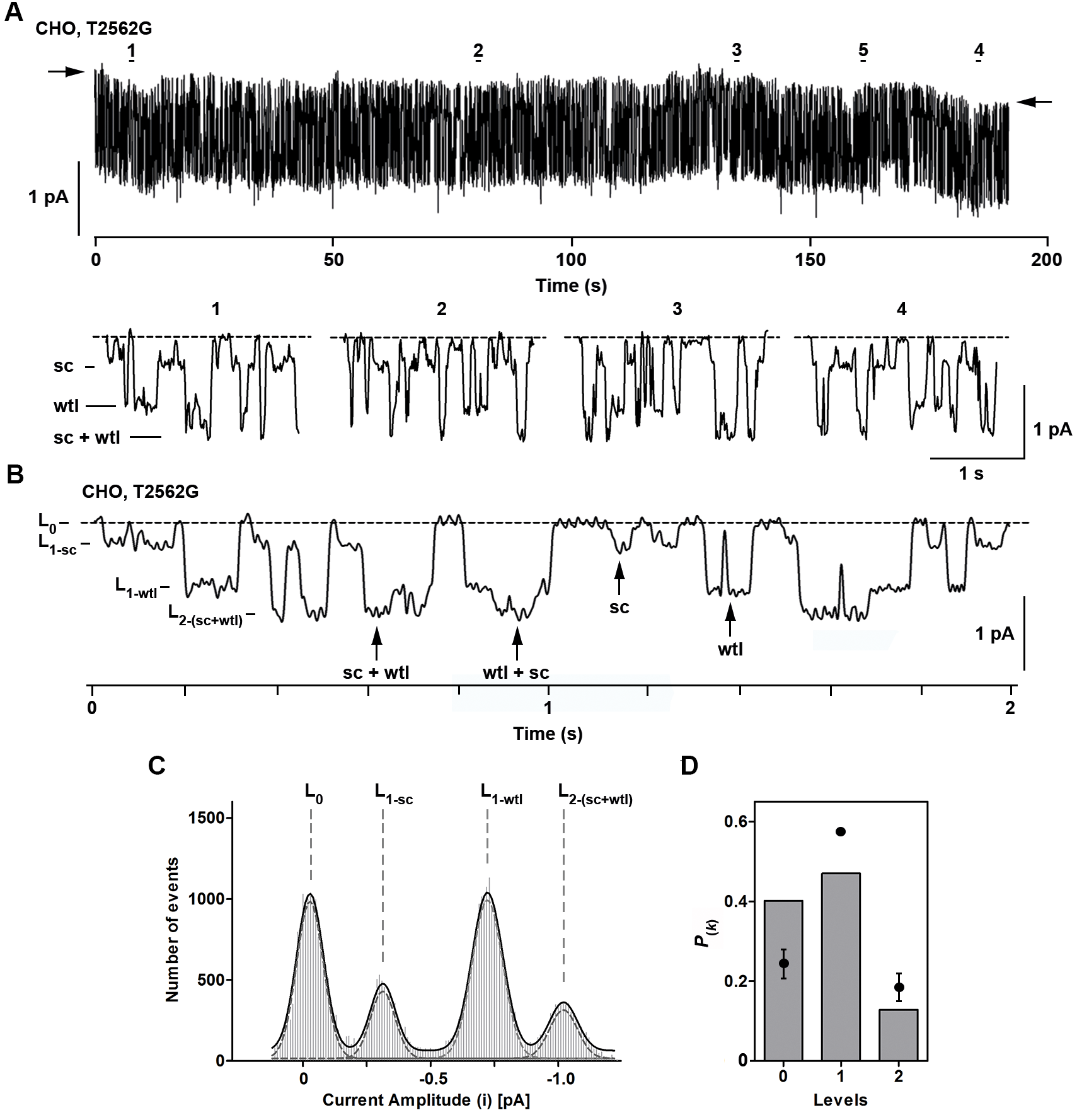

Supplement: S6 Fig — (A) A representative 3-minute single-channel recording of T2562G-CFTR in an excised inside-out membrane patch from a CHO cell commenced following full channel activation. This membrane patch contained two channels, one with sc and the other with wtl openings. ATP (1 mM) and PKA (75 nM) were continuously present in the intracellular solution. The 2-s long segments labelled 1–4 indicated by bars are displayed on an expanded time scale beneath the 3-minute recording and the segment labelled 5 indicates the high resolution recording displayed in B. Arrows and dashed lines indicate the closed channel state and downward deflections correspond to channel openings. For presentation purposes, single-channel records were filtered digitally at 50 Hz. (B-D) Binomial analysis of the T2562G channel recording displayed in A. (B) High resolution T2562G-CFTR channel recording. Letters and arrows indicate openings to different channel levels. Abbreviations: L0, both channels closed; L1-sc, one sc channel open; L1-wtl, one wtl channel open; L2-sc+wtl, one sc channel and one wtl channel open. (C) The single-channel current amplitude histogram for the T2562G-CFTR recording shown in B. The continuous line is the fit of a Gaussian distribution to the data (R = 0.94) and the dashed lines show the individual components of the Gaussian function. The vertical dashed lines indicate the positions of the different channel levels with the closed channel level (L0) shown on the left. (D) Probability (P(k)) of the wtl and sc T2562G-CFTR channels residing in either the closed level (L0), open level 1, (L1) or open level 2 (L2). Columns represent predicted P(k) values calculated using mean Po values from membrane patches containing either one wtl or one sc channel (wtl, n = 4; sc, n = 3). Symbols and error bars show experimental P(k) values calculated using Po values from three membrane patches containing one wtl channel and one sc channel. The underlying data of panel D can be found in S1 Data. (TIF [file pbio.2000779.s006.tif]

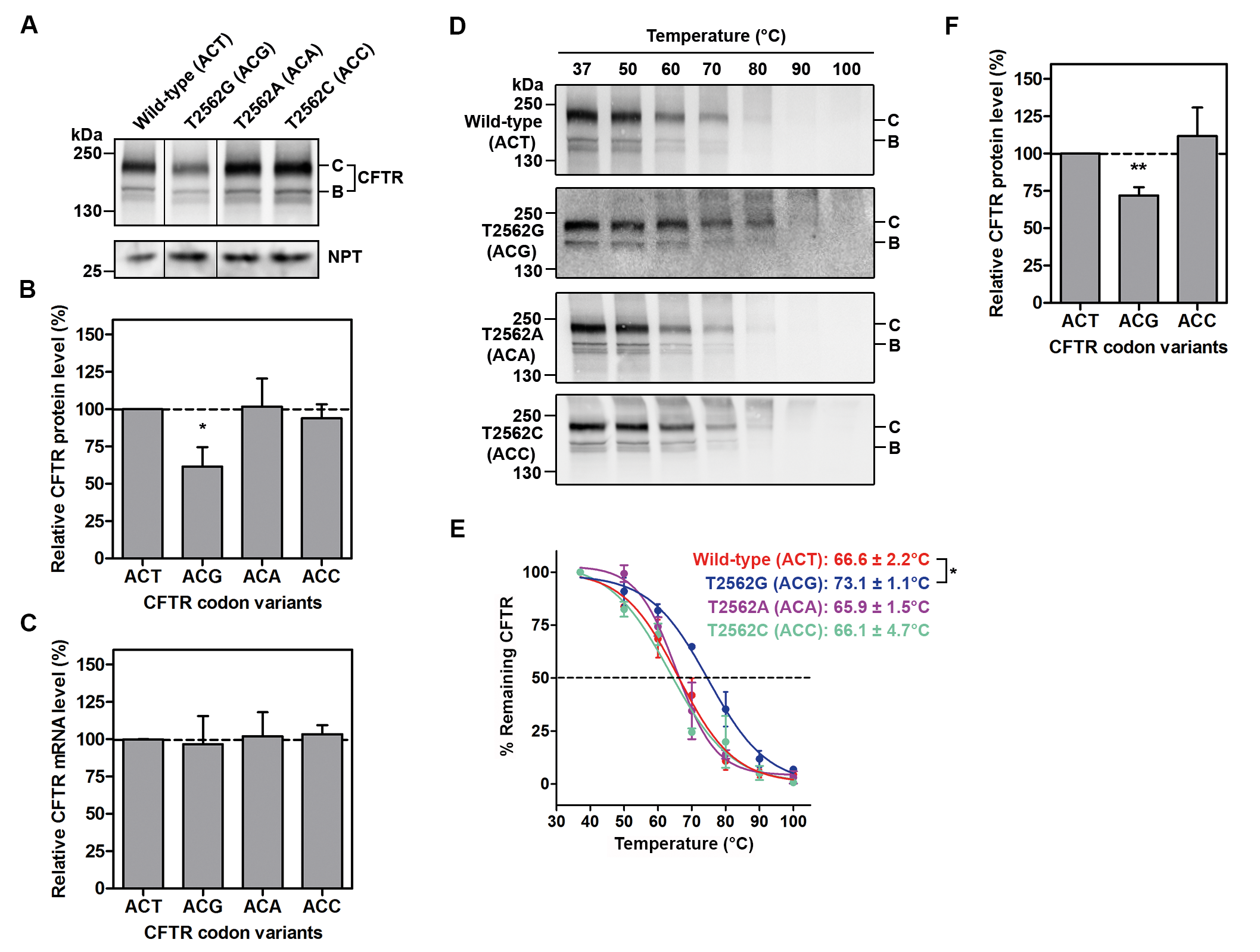

Supplement: S7 Fig — (A) Representative immunoblot analysis of CFTR T2562 sSNP variants in HeLa cells. NPT served as a transfection control. (B) Quantification of steady-state protein expression levels from the immunoblots in A. CFTR expression (i.e., the sum of bands B and C) was normalized to that of NPT to correct for differences in transfection efficiencies. The level of wild-type CFTR (ACT codon) was set to 100%. Data are means ± SEM (n = 8); * P < 0.05 versus wild-type CFTR. (C) qRT-PCR quantification of mRNA expression levels for CFTR T2562 codon variants at 24 h after transfection. The mRNA level of wild-type CFTR (ACT codon) was set to 100%. Data are means ± SEM (n = 3–7). (D, E) Thermal aggregation propensity analysis for CFTR sSNP variants. Thermal aggregation temperatures (Ta) are expressed as means ± SEM (n = 2–5); * P < 0.05 versus wild-type CFTR. (F) Quantification of the expression of CFTR codon variants in CHO cells as described in panel B. The level of wild-type CFTR was set to 100%. Data are means ± SEM (n = 4); ** P < 0.01. The underlying data of panels B-C and E-F can be found in S1 Data. (TIF) [file pbio.2000779.s007.tif]

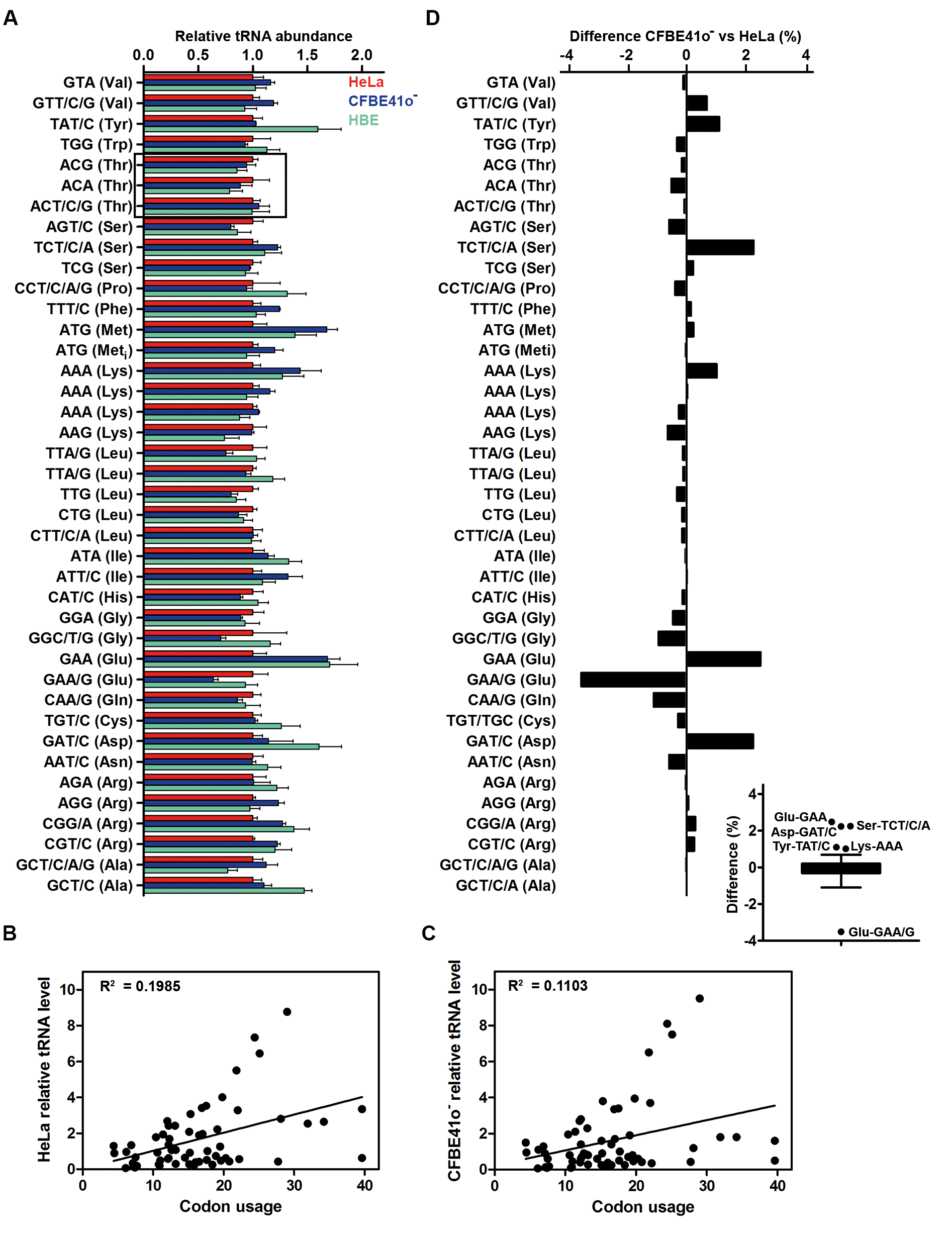

Supplement: S8 Fig — (A) Microarray analysis of relative tRNA abundance of all tRNAs in CFBE41o- and four CF patient-derived HBE cells were measured relative to the values for HeLa tRNAs. tRNA probes are depicted with their cognate codon and the corresponding amino acid; tRNAsThr highlighted by the box are presented in Fig 4D. Data are means ± SEM (n = 3–5). (B, C) Correlation between codon usage and absolute tRNA concentration for HeLa (B) and CFBE41o- cells (C). (D) Differences in tRNA abundance between CFBE41o- and HeLa cells. Negative sign denotes that the corresponding tRNAs are more abundant in CFBE41o- cells and vice versa. Inset, box-plot with designated outliers. The underlying data of panels A-D can be found in S1 Data. (TIF) [file pbio.2000779.s008.tif]

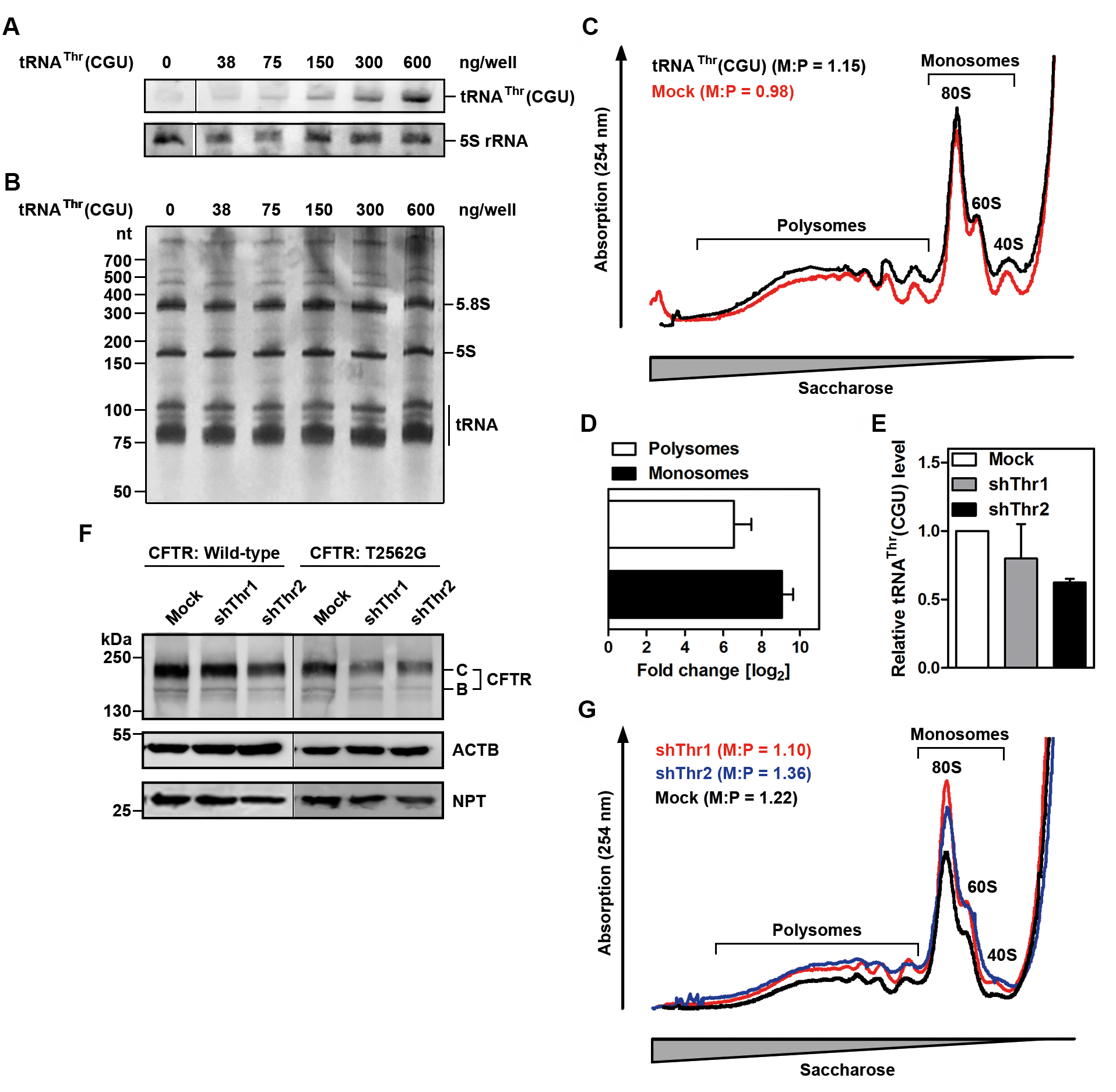

Supplement: S9 Fig — (A) Northern blot of HeLa cells transfected with increasing amounts (ng/well) of in vitro transcribed tRNAThr(CGU). 5S rRNA served as a loading control. Lane 1 (0 ng tRNA) represents nontransfected HeLa. (B) Transfection of tRNAThr(CGU) does not alter the cellular tRNA pool. (C) Addition of tRNAThr(CGU) (150 ng) does not change polysomal profiles. Mock denotes control HeLa cells. M:P denotes the ratio between monosomal (M) and polysomal (P) peaks. (D) tRNAThr(CGU) is increased in both M and P fractions. The ratio of tRNAThr(CGU) in M and P to which 150 ng tRNAThr(CGU) were added was quantified by qRT-PCR, normalized to 5S rRNA and expressed as fold-change ± SD compared to tRNAThr(CGU) levels in polysomal and monosomal fractions of untreated control HeLa (mock) cells. Values are means ± SEM (n = 3). (E) Reduction of tRNAThr(CGU) levels (reduced to ~75 or ~60%) using two shRNAs (shThr1 and shThr2), measured with qRT-PCR and normalized to 5S rRNA. Values are means ± SEM (n = 3). (F) Reduction of tRNAThr(CGU) decreases the protein levels of both wild-type and T2562G-CFTR. Representative immunoblot of four biological replicates. The positions of the band B and C forms of CFTR protein are indicated. NPT served as an internal transfection control and ACTB as a loading control. (G) Transfection with shRNAs (shThr1 and shThr2) to reduce tRNAThr(CGU) does not alter polysomal profiles. Note that tRNAThr(CGU) can only be partially downregulated as complete knockdown would perturb global translation and cell viability. The underlying data of panels D and E can be found in S1 Data. (TIF) [file pbio.2000779.s009.tif]
