# Supplementary material for: Alteration of protein function by a silent polymorphism linked to tRNA abundance
Source: PLoS Biol. 2017 May 16;15(5):e2000779. doi: 10.1371/journal.pbio.2000779 (PMC5433685; doi:10.1371/journal.pbio.2000779)
Supplement: S1 Table — (DOCX) [file pbio.2000779.s010.docx]

**S1 Table. Synonymous mutations detected in CF patients.**

| sSNP ⃰ | Amino acid | SNP ID^†^ | Codon change | ΔRSCU^‡^ |
| --- | --- | --- | --- | --- |
| G1584A^§^ | Glu 528 | rs1800095 | GAG → GAA | 0.70 |
| G2280A^§^ | Thr 760 | rs138634146 | ACG → ACA | 1.45 |
| T2562G^§^ | Thr 854 | rs1042077 | ACU → ACG | -1.40 |
| T3339C^§^ | Ala 1113 | rs1800119 | GCU → GCC | -0.53 |
| A3870G^§^ | Pro 1290 | rs1800130 | CCA → CCG | -1.07 |

⃰ Nucleotide position within the CFTR coding sequence (cDNA).

^†^ The SNP IDs for each mutation are taken from the Single Nucleotide Polymorphism database (dbSNP; www.ncbi.nlm.nih.gov/SNP).

^‡^ ΔRSCU describes the change in the relative synonymous codon usage (RSCU) for the indicated sSNP and was calculated for each mutant with the CAIcal software [1].

^§^ sSNPs were selected from the CF Mutation Database (www.genet.sickkids.on.ca/cftr/).

**References**

1. Puigbo P, Bravo IG, Garcia-Vallve S. E-CAI: a novel server to estimate an expected value of Codon Adaptation Index (eCAI). BMC Bioinformat. 2008;9: 65.
