# Supplementary material for: Alteration of protein function by a silent polymorphism linked to tRNA abundance
Source: PLoS Biol. 2017 May 16;15(5):e2000779. doi: 10.1371/journal.pbio.2000779 (PMC5433685; doi:10.1371/journal.pbio.2000779)
Supplement: S2 Table — (DOCX) [file pbio.2000779.s011.docx]

**S2 Table. Frequency of observed small-conductance openings of T2562G-CFTR.**

| Conditions | Total number of membrane patches acquired ⃰ | Membrane patches with active T2562G-CFTR channels^†^ | Membrane patches with sc openings^‡^ | Percentage of sc channels^§^ |
| --- | --- | --- | --- | --- |
| Wild-type CFTR in CHO cells | 31 | - | 1 | 3%** |
| T2562G-CFTR in HeLa cells | 35 | 13 | 7 | 54% |
| T2562G-CFTR in CHO cells | 25 | 12 | 7 | 58% |
| T2562G-CFTR in total | 60 | 25 | 14^∞^ | 56% |
| T2562G-CFTR and 150 ng tRNA^Thr^(CGU) in CHO cells | 44 | 10^‖^ | 1 | 10% |
| T2562C-CFTR in CHO cells | 26 | 9 | 0 | 0 |

⃰ Indicates the number of excised inside-out membrane patches where high GΩ membrane seals were successfully formed.

^†^ Denotes the number of excised inside-out membrane patches where active sc and wtl openings of T2562G-CFTR or channel openings of T2562C-CFTR were observed following the application of ATP (1 mM) and PKA (75 nM).

^‡^ Indicates the number of excised inside-out membrane patches that contained sc openings of T2562G-CFTR.

^§^ The number of membrane patches with sc channel openings divided by the total number of membrane patches with active T2562G-CFTR channels (%).

^∞^ Of these 14 membrane patches with sc channels, 6 were sc single-channel patches [only 3 were suitable for kinetic analyses]; 1 contained multiple sc channels; 4 contained one sc channel and one wtl channel [only 3 were suitable for kinetic analyses]; 2 contained one sc channel and multiple wtl channels and 1 contained multiple sc channels and wtl channels (i.e. 7/25 (28%) of membrane patches with active T2562G-CFTR channels only contained sc channels).

^‖^ Following tRNA^Thr^(CGU) rescue, with one exception, we only observed one population of T2562G-CFTR channels. These channels were characterised by a wild-type conductance, but a slightly reduced open probability.

**Small-conductance-like openings of wild-type CFTR are very rare events and distinct from the sc openings of T2562G-CFTR. For wild-type CFTR, they represent very rare examples of mode switching, whereas for T2562-CFTR, when sc openings are observed they persist for the entire duration of single-channel recordings either by themselves or together with wtl openings of T2562G-CFTR.
