# Supplementary material for: Alteration of protein function by a silent polymorphism linked to tRNA abundance
Source: PLoS Biol. 2017 May 16;15(5):e2000779. doi: 10.1371/journal.pbio.2000779 (PMC5433685; doi:10.1371/journal.pbio.2000779)
Supplement: S3 Table — (DOCX) [file pbio.2000779.s012.docx]

**S3 Table. Frequency of usage of different codons in CFTR compared to their usage in the human genome.**

The codons encoding Thr are highlighted in gray.

| Amino acid | Codon | *H. sapiens* ⃰ | CFTR | |
| --- | --- | --- | --- | --- |
|  |  | /1000^†^ | /1000^†^ | RSCU^‡^ |
| Gly | GGG | 16.47 | 10.8 | 0.76 |
|  | GGA | 16.47 | 25.66 | 1.81 |
|  | GGT | 10.75 | 10.13 | 0.71 |
|  | GGC | 22.22 | 10.13 | 0.71 |
| Glu | GAG | 39.59 | 19.58 | 0.65 |
|  | GAA | 28.96 | 43.21 | 1.35 |
| Asp | GAT | 21.78 | 25.66 | 1.31 |
|  | GAC | 25.10 | 13.50 | 0.69 |
| Val | GTG | 28.12 | 24.31 | 1.62 |
|  | GTA | 7.08 | 8.10 | 0.54 |
|  | GTT | 11.03 | 15.53 | 1.03 |
|  | GTC | 14.46 | 12.15 | 0.81 |
| Ala | GCG | 7.37 | 4.05 | 0.29 |
|  | GCA | 15.82 | 22.96 | 1.64 |
|  | GCT | 18.45 | 18.23 | 1.30 |
|  | GCC | 27.73 | 10.80 | 0.77 |
| Arg | AGG | 11.96 | 10.80 | 1.23 |
|  | AGA | 12.17 | 24.98 | 2.85 |
| Ser | AGT | 12.13 | 12.83 | 0.93 |
|  | AGC | 19.46 | 16.21 | 1.17 |
| Lys | AAG | 31.86 | 23.63 | 0.76 |
|  | AAA | 24.44 | 38.49 | 1.24 |
| Asn | AAT | 16.96 | 17.56 | 0.93 |
|  | AAC | 19.10 | 18.91 | 1.07 |
| Met | ATG | 22.04 | 25.66 | ­ – |
| Ile | ATA | 7.49 | 22.28 | 0.83 |
|  | ATT | 16.00 | 34.44 | 1.26 |
|  | ATC | 20.82 | 23.63 | 0.91 |
| Thr | ACT | 13.12 | 21.61 | 1.54 |
|  | ACG | 6.05 | 2.03 | 0.14 |
|  | ACA | 15.11 | 22.28 | 1.59 |
|  | ACC | 18.89 | 10.13 | 0.72 |
| Trp | TGG | 13.17 | 15.53 | ­ – |
| Cys | TGT | 10.58 | 5.40 | 0.89 |
|  | TGC | 12.62 | 6.75 | 1.11 |
| Tyr | TAT | 12.19 | 14.85 | 1.10 |
|  | TAC | 15.31 | 12.15 | 0.90 |
| Leu | TTG | 12.93 | 22.96 | 1.11 |
|  | TTA | 7.67 | 24.98 | 1.21 |
| Phe | TTT | 17.57 | 31.74 | 1.11 |
|  | TTC | 20.28 | 25.66 | 0.89 |
| Ser | TCG | 4.41 | 2.70 | 0.20 |
|  | TCA | 12.21 | 19.58 | 1.41 |
|  | TCT | 15.22 | 20.26 | 1.46 |
|  | TCC | 17.68 | 11.48 | 0.83 |
| Arg | CGG | 11.42 | 5.40 | 0.62 |
|  | CGA | 6.17 | 6.08 | 0.69 |
|  | CGT | 4.54 | 2.03 | 0.23 |
|  | CGC | 10.42 | 3.38 | 0.38 |
| Gln | CAG | 34.23 | 20.93 | 0.93 |
|  | CAA | 12.34 | 24.31 | 1.07 |
| His | CAT | 10.86 | 7.43 | 0.88 |
|  | CAC | 15.09 | 9.45 | 1.12 |
| Leu | CTG | 39.64 | 24.98 | 1.21 |
|  | CTA | 7.15 | 12.83 | 0.62 |
|  | CTT | 13.19 | 21.61 | 1.05 |
|  | CTC | 19.59 | 16.21 | 0.79 |
| Pro | CCG | 6.92 | 0.68 | 0.09 |
|  | CCA | 16.92 | 8.78 | 1.16 |
|  | CCT | 17.54 | 13.50 | 1.78 |
|  | CCC | 19.79 | 17.43 | 0.98 |

*⃰ H. sapiens* codon usage table was downloaded from the Codon Usage Database (www.kazusa.or.jp/codon).

^†^ Defined as frequency per thousand codons. Codon usage of human CFTR was calculated with the Sequence Manipulation Suite (www.bioinformatics.org/SMS/index).

^‡^ Relative synonymous codon usage (RSCU) was calculated using the CAIcal software [1].

**References**

1. Puigbo P, Bravo IG, Garcia-Vallve S. E-CAI: a novel server to estimate an expected value of Codon Adaptation Index (eCAI). BMC Bioinformat. 2008;9: 65.
