# Supplementary material for: Alteration of protein function by a silent polymorphism linked to tRNA abundance
Source: PLoS Biol. 2017 May 16;15(5):e2000779. doi: 10.1371/journal.pbio.2000779 (PMC5433685; doi:10.1371/journal.pbio.2000779)
Supplement: S1 Text — (DOCX) [file pbio.2000779.s013.docx]

Variation in tRNA abundance between different cells

On the tRNA-specific microarrays, tRNAs are detected by full-length tDNA probes [[1](#_ENREF_1)]. This approach reliably distinguishes between tRNA species with at least eight nucleotides difference [[1](#_ENREF_1)], so that from all human tRNAs we quantified 37 out of the 49 different isoacceptors. HeLa and CFBE41o^-^ cells were dissimilar in 7 tRNAs (S8D Fig.). Compared to the primary CF-patient-derived HBE cells, the total concentration of all tRNAs was higher in CFBE41o^-^ cells, but the proportion of each single tRNA within the whole tRNA pool was very similar among them (S8A Fig.). Only two tRNAs were significantly higher in primary CF-patient-derived HBE cells than CFBE41o^-^ cells: alanine tRNA reading GCT and GCC codons and tyrosine tRNA pairing to TAT and TAC codons (S8A Fig.). The three tRNAs reading the four Thr codons exhibited similar levels in HeLa, CFBE41o^-^ and primary HBE cells (Fig. 4D, E and S8A Fig.).

When using comparative microarrays (Fig. 4D, G and S8A Fig.) each tRNA isoacceptor of a cell line or tissue is compared to one cell line, usually one for which absolute quantitative data exist, in this case HeLa cells (Fig. 4C). Thus, a ratio of one means that the concentration of this isoacceptor is equal to HeLa, whereas ratio higher or smaller than one means that this isoacceptor is at concetration higher or lower than the one in HeLa cells (Fig. 4D, G and S8A Fig.).

**Supporting Materials and Methods**

Assessment of T2562G-driven alternative splicing of the CFTR transcript

The assay was perfomed according to previous publications, which evaluated mutation-based exon skipping in CFTR [[2-4](#_ENREF_2)]. Briefly, exon 15 from wild-type or T2562G-CFTR was amplified with the flanking intronic regions, e.g. 294 bp upstream and 210 bp downstream, using the following primers: 5’-ATAAATCTCGAGGCCTTTAATGGTAAAATTGTCC-3’ and 5’-ATAAATGCGGCCGCGAATCTATACCATTATATACTG-3’. The PCR product was double digested with *Xho*I and *Not*I Fast Digest restriction enzymes and cloned into pET01 vector (Mobitec) [[5](#_ENREF_5)]. Equal amounts of each construct, i.e. wild-type or T2562G-CFTR exon 15-containing pET01, was transfected into CFBE41o^-^ cells. 24 h after transfection, total RNA was extracted using TRI Reagent. 1.5 µg of total RNA was DNaseI treated for 30 min and heat inactivated at 65°C. 400 ng of the total DNaseI-treated RNA was used for cDNA synthesis with transcript-specific RT primers using Revert Aid H Minus M-MuLV Reverse Transcriptase. Two µl of each cDNA were amplified using primers specific to the splice acceptor and donor within the pET01 plasmid (5’-GAGGGATCCGCTTCCTGGCCC-3’ and 5’-CTCCCGGGCCACCTCCAGTGCC-3’). PCR products were purified (Thermo Scientific PCR clean up Kit) and the product size was analyzed on agarose gel and on a Bioanalyzer using DNA1000 Chip (Agilent).

Detection of ubiquitinylated CFTR and co-immunoprecipitation

Cells expressing various CFTR proteins for 24 h were pelleted at 500×g for 10 min at 4°C and subsequently lysed in co-immunoprecipitation (coIP) buffer (50 mM Tris-HCl , pH 7.5, containing 50 mM NaCl, 0.5% NP-40, 10% glycerol and 1× complete protease inhibitor) buffer supplemented with 10 mM N-ethylmaleimide (NEM, Sigma-Aldrich) for 30 min on ice. Cell debris was removed by centrifugation for 5 min at 14,000×g (4°C) and the supernatant corresponding to 0.5 - 1 mg of total protein in 0.5 ml total volume was used for coIP. For coIP of CFTR-bound proteins, 4 µl mouse anti-CFTR R-domain antibody (570) was added and samples were incubated overnight at 4°C with gentle agitation. Magnetic µMACS protein G MicroBeads were added for a further 6 h and immunocomplexes were separated using µColumns. After washing the µColumns 4× with coIP buffer, bound proteins were eluted with 2× SDS-loading buffer pre-heated to 50°C. Equal amounts of eluate were loaded onto SDS gels and proteins detected by immunoblotting using rabbit anti-CHIP (dilution 1:1000; Sigma-Aldrich, no. C9243); or rabbit anti-RMA1 (dilution 1:1500; Sigma-Aldrich, no. SAB4200208). For quantification, intensities of bound CHIP and RMA1 were normalized to the amount of precipitated CFTR (i.e. sum of bands B and C). To detect ubiquitinylated CFTR protein, HeLa cells were co-transfected with CFTR constructs and HA-ubiquitin-GFP [[6](#_ENREF_6)] and incubated for 6 h with 10 µM MG132 and subjected to co-immunoprecipitation with mouse anti-CFTR R-domain antibody (570) overnight at 4°C, as described above; ubiquitinylated CFTR proteins were detected by immunoblotting using mouse anti-ubiquitin-HRP (dilution 1:200; Santa Cruz Biotechnology, no. SC-8017).

Pulse-chase analysis

For pulse-chase assays, HeLa cells were washed once with Hank's Balanced Salt Solution (HBSS; Gibco) and incubated in starvation medium without cysteine (Cys) and methionine (Met) (MP Biomedicals) for 20 min at 37°C. Pulse-labeling was performed in medium containing ^35^S-Cys and ^35^S-Met (both 200-250 nCi/µl) for 15 min. Incorporation of radiolabeled Met and Cys was stopped by adding chase medium containing 50 mM unlabeled Cys and Met. All media contained and 10 mM HEPES to maintain buffering capacity. After different chase times, the cells were transferred to ice, washed twice with cold HBSS and lysed using MNT buffer containing 0.5% Triton X-100. Radiolabeled CFTR protein was immunoprecipitated using polyclonal anti-CFTR NBD1 antibody (Mr. Pink) at 4°C overnight, resolved on SDS-PAGE and the intensities of bands B and C were determined by autoradiography (Typhoon FLA-700 with ImageQuant software for analysis, GE Healthcare). To quantify data, the intensity of band B at 0 min of the chase for a CFTR construct was set to 1 and the intensities of bands B and C at all other chase times were expressed relative to this zero-time value for band B.

Partial permeabilization of cells and limited proteolysis

Semi-intact (SI) cells were prepared as described previously [[7](#_ENREF_7)]. HeLa cells were harvested using 0.05% trypsin-EDTA (Gibco) and washed once in cold KHM [[7](#_ENREF_7)] buffer (110 mM KOAc, 20 mM HEPES-KOH, pH 7.2, 2 mM MgOAc). Cells were resuspended in 6 ml ice-cold KHM buffer containing 6 µl of digitonin (40 mg/ml stock dissolved in DMSO; Merck) and incubated for exactly 5 min on ice. Permeabilization was stopped by adding 8 ml KHM buffer and pelleting the cells by centrifugation at 4°C. SI cells were washed, resuspended in KHM buffer and stored at -80°C.

The protease susceptibility of CFTR constructs was determined using limited proteolysis. For limited proteolysis, SI cells corresponding to 75 µg protein lysate were placed on ice and trypsin (TPCK treated, dissolved in 1 mM HCl to 10 mg/ml stock; Sigma-Aldrich) added in concentrations ranging from 37.5 µg/ml to 0.075 µg/ml. After 15 min incubation on ice, the reaction was stopped by adding 2.5× complete protease inhibitor and 2× SDS-loading buffer. Samples were heated to 37°C for 10 min and centrifuged for 5 min at 17,000×g (4°C) to remove cell debris. The supernatant was mixed with 12.5 U benzonase nuclease (Novagen), incubated for 15 min on ice and samples were run on a 10% SDS-PAGE. Proteolytic fragments were detected by immunoblotting using the anti-CFTR NBD1 (660) antibody.

Polysome profiling

Polysomes were isolated as follows: Cells were treated for 10 min at 37°C with 100 µg/ml cyclohexamide (CHX; Sigma), harvested by trypsinization, resuspended in ice-cold polysome lysis buffer (10 mM Tris-HCl, pH 7.4, containing 5 mM MgCl_2_, 100 mM KCl, 1% Triton X-100, 2 mM DTT and 100 µg/ml CHX) and lysed with 8 strokes of a 26-gauge needle. Cell debris was removed by centrifugation at 2,500×g for 8 min (4°C) and equal amounts of supernatant (adjusted to absorption at 260 nm) were applied onto a 15% to 50% (w/v) saccharose (Roth) gradient (20 mM HEPES-KOH, pH 7.4, supplemented with 5 mM MgCl_2_, 100 mM KCl, 100 µg/ml CHX and 2 mM DTT). After centrifugation for 1.5 h at 35,000 rpm (4°C) in a SW55Ti Rotor (Beckman Coulter), gradients were analyzed from bottom to top by absorption measurement at 254 nm using a flow-through UV spectrophotometer cell (UV-M II UV detector; Pharmacia Biotech). Monosomal and polysomal peak areas were determined using Prism 5 software.

**References**

1. Dittmar KA, Goodenbour JM, Pan T. Tissue-specific differences in human transfer RNA expression. PLoS Genet. 2006;2: e221.

2. Aissat A, de Becdelievre A, Golmard L, Vasseur C, Costa C, Chaoui A, et al. Combined computational-experimental analyses of CFTR exon strength uncover predictability of exon-skipping level. Hum Mutat. 2013;34: 873-81.

3. Masvidal L, Igreja S, Ramos MD, Alvarez A, de Gracia J, Ramalho A, et al. Assessing the residual CFTR gene expression in human nasal epithelium cells bearing CFTR splicing mutations causing cystic fibrosis. Eur J Hum Genet. 2014;22: 784-91.

4. Steiner B, Truninger K, Sanz J, Schaller A, Gallati S. The role of common single-nucleotide polymorphisms on exon 9 and exon 12 skipping in nonmutated CFTR alleles. Hum Mutat. 2004;24: 120-9.

5. Hinzpeter A, Aissat A, Sondo E, Costa C, Arous N, Gameiro C, et al. Alternative splicing at a NAGNAG acceptor site as a novel phenotype modifier. PLoS Genet. 2010;6, e1001153.

6. Steffen J, Seeger M, Koch A, Kruger E. Proteasomal degradation is transcriptionally controlled by TCF11 via an ERAD-dependent feedback loop. Mol Cell. 2010;40: 147-58.

7. Kleizen B, van Vlijmen T, de Jonge HR, Braakman I. Folding of CFTR is predominantly cotranslational. Mol Cell. 2005;20: 277-87.
